# Macroporous Semiconductors

**DOI:** 10.3390/ma3053006

**Published:** 2010-05-07

**Authors:** Helmut Föll, Malte Leisner, Ala Cojocaru, Jürgen Carstensen

**Affiliations:** Institute for Materials Science, Christian-Albrechts-University of Kiel, Kaiserstr. 2, D-24143 Kiel, Germany; E-Mails: ml@tf.uni-kiel.de (M.L.); ac@tf.uni-kiel.de (A.C.); jc@tf.uni-kiel.de (J.C.)

**Keywords:** porous semiconductors, macropores in semiconductors, electrochemistry of semiconductors, *in situ* impedance spectroscopy, applications of porous semiconductors, modeling of pore formation

## Abstract

Pores in single crystalline semiconductors come in many forms (e.g., pore sizes from 2 nm to > 10 µm; morphologies from perfect pore crystal to fractal) and exhibit many unique properties directly or as nanocompounds if the pores are filled. The various kinds of pores obtained in semiconductors like Ge, Si, III-V, and II-VI compound semiconductors are systematically reviewed, emphasizing macropores. Essentials of pore formation mechanisms will be discussed, focusing on differences and some open questions but in particular on common properties. Possible applications of porous semiconductors, including for example high explosives, high efficiency electrodes for Li ion batteries, drug delivery systems, solar cells, thermoelectric elements and many novel electronic, optical or sensor devices, will be introduced and discussed.

## Table of Contents

1. Introduction3007 1.1. Scope of the Paper3007 1.2. Some Pictures and Definitions30072. Pore Etching Experiments3012 2.1. Etching System and Degrees of Freedom3012 2.2. Current and Dissolution3016 2.3. First Insight into Pore Formation30173. Some General Aspects of Pores in Semiconductors3022 3.1. Major Differences and Common Features3022 3.2. Self Organization and Pore Growth Mode Transitions3025 3.3. Pore Geometry3030 3.4. A Closer Look at Self-Organization Issues3033 3.5. A Closer Look at Growing Deep Pores30364. Multi-Mode-*In-Situ* FFT Impedance Spectroscopy During Pore Etching3038 4.1. General Technique3038 4.2. Selected Results from Si Pore Etching3043 4.3. A Case Study for InP and GaAs3046  4.3.1. Studying Crysto and Curro Pores in InP3046  4.3.2. Crysto and Curro Pores in GaAs30545. Some Applications of Porous Semiconductors3056 5.1. General Remarks3056 5.2. Some New Applications3060  5.2.1. Sensors Exploiting the Piezoelectric Properties of Porous III-V3060  5.2.2. Single Holes with Large Aspect Ratios3060  5.2.3. Anodes for Li Ion Batteries30606. Summary and Conclusions3062Acknowledgements3063References and Notes3063

## 1. Introduction

### 1.1. Scope of the Paper

Doing justice to all aspects of “Porous Semiconductors” would require a good-sized book, if not a whole series of books. In fact, several books about the topic have already been written. Most important in this context are perhaps the monographs of Zhang [1] and Lehmann [2], dealing mostly with the electrochemistry of semiconductors and with pore etching in Si, respectively. Then we have the by now five proceedings of the bi-annual International Conference on Porous Semiconductors Science and Technology (PSST) [3,4,5,6,7] and some special proceedings volumes of the Electrochemical Society [8,9,10]. Kochergin and Föll published a recent monograph [11] focusing on optical properties of mostly porous Si, and Notten, van den Meerakker, and Kelly looked into the general electrochemistry of III-V semiconductor compounds some years ago [12]. There is also no lack of reviews. In [13,14,15,16,17,18,19,20,21,22] many aspects of pores in semiconductors are highlighted; more recent reviews deal with macropores in Si [23], self-organization issues in the context of pore formation in semiconductors [24], the luminescence from porous Si [18,25,26,27], or the use of impedance techniques for *in situ* assessment of pore growth [28].

Reading through the several thousand pages quoted above will not only provide a lot of information about porous semiconductors (even more can be found in the by now several thousand regular papers), but will also illustrate two pertinent points: i) there are still many open questions concerning, e.g., formation mechanisms and properties of porous semiconductors, and ii) there has been much progress in theory, techniques, and experimental discoveries in recent years. To give just a few examples for the latter point: pore types completely different from anything else encountered so far were discovered in GaP in [29], in Ge in 2009 [30], and in ZnSe in [31,32]. The fact that these pore types were neither predicted nor understood at present, nicely illustrates the first point.

Accordingly, in this review we must restrict ourselves to some major aspects of pores in some semiconductors and omit many details. The first restriction is that this review deals mostly with macropores, *i.e.*, pores with diameters >50 nm. For the smaller meso and micropores the reader is referred to the recent review of Sa’ar [25], the forthcoming book of Sailor [33], and the references therein. What will be dealt with here are i) some general aspects of macropore formation in semiconductors, *i.e.*, pore properties found in several semiconductors, ii) some general aspects of pore formation models with particular emphasize on stochastic models, iii) the more recently employed techniques of *in situ* measurement techniques during pore formation, and iv) a short overview of special properties of porous semiconductors with respect to potential applications. All points will be illustrated in some detail with old but also quite recent results mostly from the Kiel (Germany) group of the authors.

### 1.2. Some Pictures and Definitions

**Figure 1:**
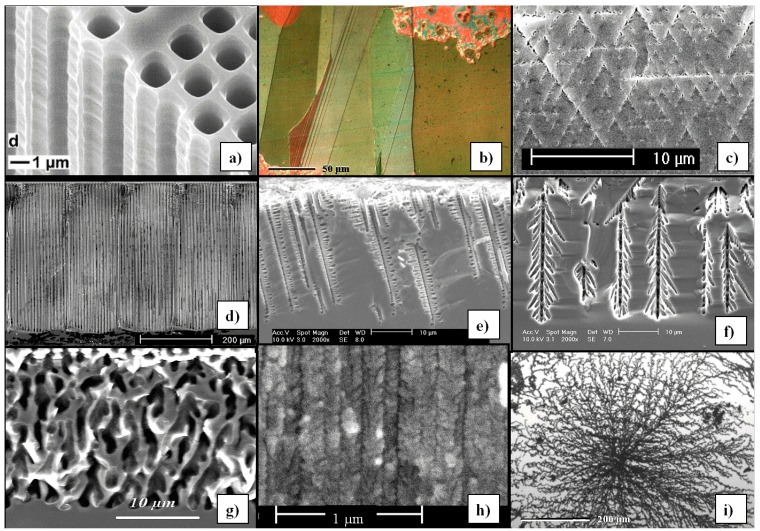
Examples of pores encountered in anodically etched Silicon; (b) and (i) are top views, all others are SEM cross-sections. (a) n-macro(bsi (backside illumination), aqu, litho); (b) microporous layer on poly-crystalline Si with interference colors, (c) n-macro(bsi, aqu, litho, {111}); pores grow in <113> directions, (d) deep n-macro(bsi, aqu, litho); (e) n-macro(fsi (frontside illumination), org; off-{100}); (f) n-macro(bsi, org); (g) p-macro(org); (h) n-meso(aqu); (i) fractal surface “pores” in n-Si at extremely low HF concentration. “aq” and “org” refer to aqueous or organic electrolyte; the later usually containing DMSO or DMF. For details refer to the text and [13,35,36,37,38,39,40].

**Figure 2:**
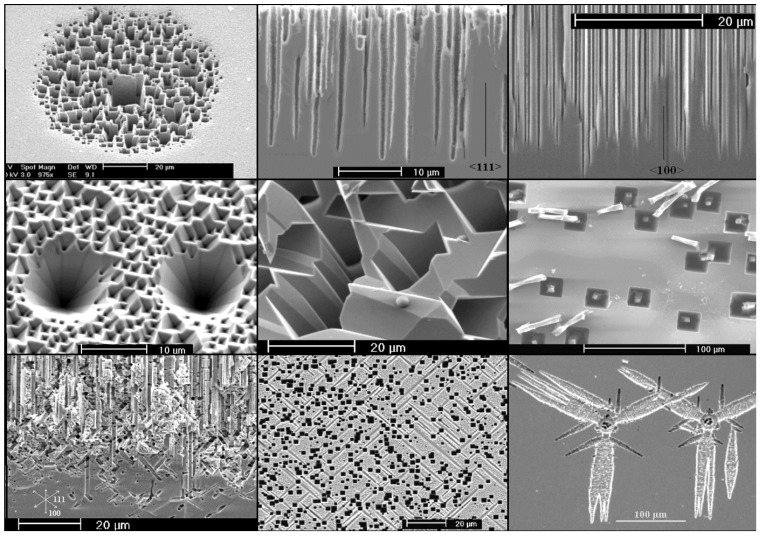
Example of pores encountered in anodically etched Ge. n-type and p-type Ge is covered; for details see [48,49,50,51,52,53,54].

**Figure 3:**
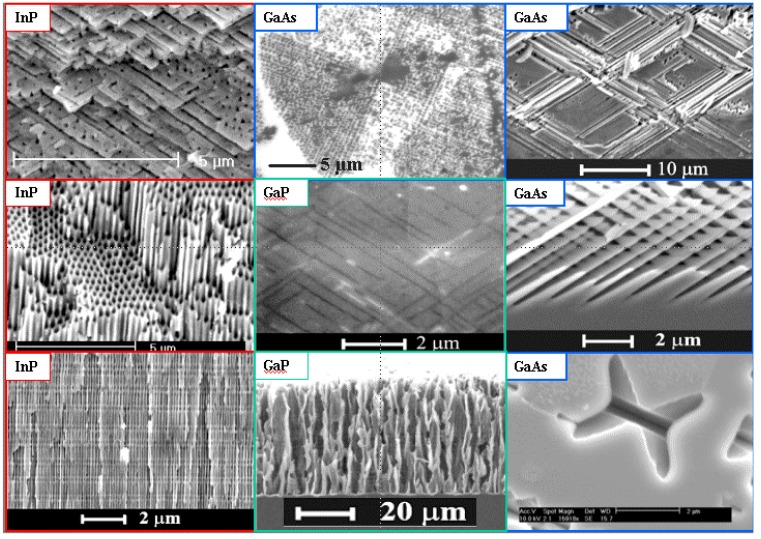
Example of pores encountered in anodically etched III-V’s as indicated. Pores can only be generated in n-type. For details see [14,55,56,57,58,59,60,61,62,63].

A certain problem when dealing with pores in semiconductors is the terminology. It is hard to describe the structure of porous semiconductors in words because we encounter a huge range of geometries and morphologies, Figure 1, Figure 2 and Figure 3 give a flavor of this. The only (not very helpful) formal definition considers only the pore geometry in the sense of the (average) pore size (the word pore diameter is already too special because it implies a circular cross-section), and possibly the (average) distance between pores. According to an IUPAC convention [34], we distinguish geometrically between micropores (diameter < 2 nm), mesopores (diameter 2 nm–50 nm), or macropores (diameter > 50 nm). However, the formally undefined term “nanopores” is also used a lot for what formally should be termed micro- or mesopores. There is no convention on the use of the term “morphology”, meaning the three-dimensional appearance of pores, and there are many ad-hoc terms that relate to this like: branched pores, tree-like pores, nano-sponge; crystallographic pores, current line pores, and so on. Perfectly straight cylindrical pores are found as well as highly disordered two- or three-dimensional fractal arrangements - and anything conceivable in between. In this paper we will stick to the IUPAC convention if not otherwise noted.

Figure 1, Figure 2 and Figure 3 show exclusively pore types found in the group of the authors because these 27 pictures suffice to give a taste of what “pores in semiconductors” imply. They also demonstrate that a paper endeavoring to deal with all kinds of pores identified so far would be long and tedious. There are even more pore types than shown and the authors could add copiously to the list - and so could many other groups active in the field. Besides pores in the five semiconductors shown in the figures there are also specific pores found in, e.g., SiC (cf. [41,42,43,44,45,46,47]) or in II-VI materials like ZnSe (cf. [31]), and the reader should be aware of this.

Considering pores in semiconductors it is not only necessary to define the pore geometry and morphology in a sensible way - it is also imperative to define the basic parameters of the semiconductor being etched, and pertinent details of the electrochemical processing. Besides the semiconductor itself, it is important to give its doping type (p-type or n-type) and at least a rough idea of the doping level (e.g., p or p^+^; there are often distinct differences between lightly or heavily doped semiconductors) and the crystallographic orientation (including “A” or “B” direction for polar directions; e.g., {111}A for III-V’s, if the surface terminates in group III atoms) of the samples used. It is customary, however, to omit the latter point if the standard {100} orientation was used. In some cases (essentially Si and Ge) the use of illumination is essential, distinguished by either front side illumination (fsi) or backside illumination (bsi). The nucleation process of the pores plays a large role (something not yet fully appreciated or understood); at the very minimum we must distinguish extrinsic nucleation, usually introduced by employing some lithographically structured nucleation layer (and therefore abbreviated “litho” here), and intrinsic nucleation, *i.e.*, nucleation that happens “somehow” (abbreviated “random” if necessary) as essential part of the pore etching process. Pore etching proceeds either under galvanostatic (*i.e.*, constant current) or potentiostatic (*i.e.*, constant electrode potential) conditions, or under more complex external conditions that may change with time. Some electrolyte is used that may have a rather special composition at some controlled or uncontrolled (“ambient”) temperature.

It is impractical to go through the full list for every example. In order to provide the absolutely necessary minimum of information, while still providing easy readability, we adopt the mostly self-explaining nomenclature introduced in [13] and refer, for example, to (straight) macropores in {100} n-type Si obtained by employing back side illumination in an aqueous electrolyte and with lithographically provided nucleation sites as: n-Si-macro(aqu, bsi, litho) pores. Not mentioned are the usual standards and specialties: (controlled) room temperature, typically galvanic conditions, electrolyte circulation, starting conditions different from steady state, and so on.

Looking at the 27 examples provided demonstrates some noteworthy common characteristic: it is quite obvious that pores often (but not always) express the crystallography of the substrate by preferring certain directions for growth. This may not be so remarkable for the typically found <100> directions in Si and Ge, or the <111>B directions in III-V’s - but why <113> should be a preferred direction in Si [61,62,64] is still rather mysterious.

Moreover, while often a high degree of order is found, some “stochastics” is clearly present, too. The first point is expressed most spectacularly in the pore crystal visible for the InP case in Figure 3 (left, middle) but also in the rather regular arrangement of crossing pores for the GaAs case in Figure 3 (right, middle) or in the rather regular pore branching in Figure 1e), Figure 1f). The fractal pores in Figure 1i) demonstrate pure stochastics in the sense that a growing pore tip will grow in any available direction with about equal probability. Even the most regular structures found (and seeded without lithography) still exhibit stochastic properties in the sense that there is still some randomness with respect to geometric and morphological parameters.

It is important to keep in mind that all the pictures shown present a snapshot taken *ex-situ* or “post mortem” at the time the experiment was stopped, and that the picture may not contain much information about how the observed state was achieved as a function of time, nor how pore growth would proceed. To be sure, in most experiments there is a region in time and space, where the pores grow more or less “monotonously”, *i.e.*, the structure would look about the same except for the length of the pores but that is neither true for short times, nor for long times. Common sense dictates that pores cannot grow indefinitely into the depth of the sample, since moving the necessary chemical species down to the tip and the reaction products out of the pore becomes increasingly difficult, not to mention that the potential at the pore tip drops continuously because of ohmic losses down the length of the pore. Pore growth as a function of time is indeed a complex issue, in particular because parameters often do not just change gradually but often suddenly—a pore growth mode transition is observed, cf. [65].

It is thus obvious that there cannot be a simple model that produces the observed pore characteristics as a function of time by just “plugging in” the major external parameters, *i.e.*, semiconductor data, electrolyte data, and system data. It is equally obvious that those data, if complete, do determine everything that results. One problem is that nobody knows for sure what exactly would constitute a complete parameter set. In other words, how important are often ignored fine details, e.g., the exact cell design determining the exact flow pattern of the electrolyte or a crystallographic direction of the sample surface relative to the flow direction of the electrolyte? There are indications that the examples mentioned are important on occasion and that simply means that any sufficiently sophisticated general pore formation model is still far beyond the present state-of-the art.

However, it becomes increasingly clear that behind the staggering multitude of pores in semiconductors some general common “meta” features can be found in many circumstances. This indicates that on a suitable meta-level description some “simple” principles might operate that are independent of “details” like the exact chemical process of dissolution for a given semiconductor in a given chemistry. In this paper we endeavor to elucidate these general principles as far as that is presently possible.

## 2. Pore Etching Experiments

### 2.1. Etching System and Degrees of Freedom

**Figure 4:**
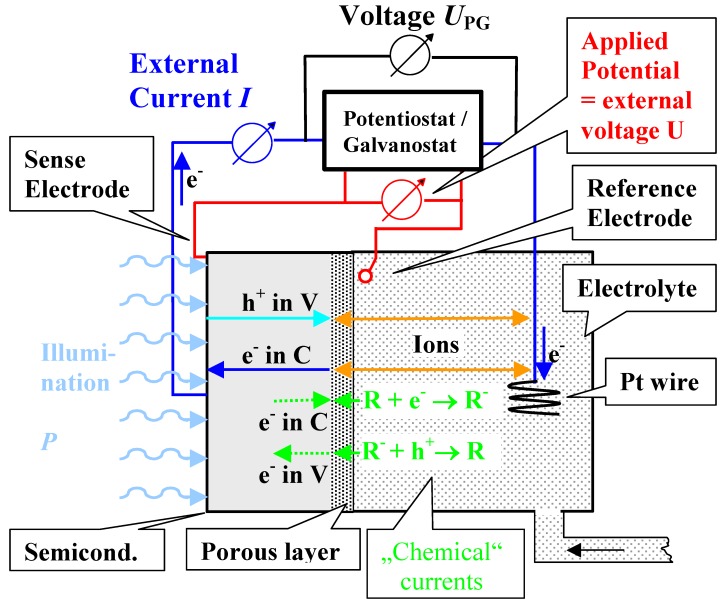
Principles of etching apparatus and current flow upon anodically dissolving n-type Si. Note the separation into external currents and “chemical” currents.

Many R&D groups are etching pores in semiconductors, cf. [3,4,5,6,7]. Most groups use small samples of about (3–10) mm^2^ employing some experimental set-up that is typically self-build with respect to the electrolytical cell and the electrolyte handling, because that part of the experimental set-up seems to be simple. While this is true enough in principle, the results of a pore etching experiment may sensitively depend on details of the experimental set-up since the dissolution process is on occasion quite sensitive to some of the experimental details, like pumping of the electrolyte or small changes of the electrolyte composition. Figure 4 illustrates a few relevant points in this context.

Shown is the typical set-up for etching n-Si-macro(aqu, bsi, litho) pores first described by Lehmann and Föll in 1990 [66], the kind of pores that might be considered the paragon of “pores in semiconductors” next to the luminescent p-Si-micro(aqu) pores discovered by Canham [67] and Lehmann and Gösele [68] in 1991.

The back side illumination (bsi) with an intensity or power *P* generates electron-hole pairs near the back side surface; some of the holes (= minority carriers) now situated in the valence band of the Si (h^+^ in V), after going for some random walk, will reach the reactive Si—electrolyte interface, trigger the dissolution reaction, and become part of the external current. Other holes are lost for this process by recombination somewhere else. The reaction sequence thus initiated may lead to the injection of electrons into the conduction band (e^-^ in C), and both the electron and the hole current in the Si are converted to an external extrinsic current *I* flowing across the potentiostat/galvanostat (PG), that defines the external electrical parameters. Purely chemical reactions may also occur as indicated, leading to a “chemical current” that changes the chemistry (e.g., by H_2_ evolution) and is not part of the external current; cf. for example [11]. Since substantial voltage drops may be encountered in the electrolyte and the periphery, it is good practice, if not absolutely required, to refer to the potential with respect to a reference electrode near the interface and a voltage probe (usually called sense electrode) at the sample back side. The electrolyte often needs to be circulated to avoid concentration gradients at the interface and should be stabilized with respect to temperature.

While this is still rather general and simple, the complications start as soon as we consider the particulars of a more difficult case: large area etching of Si, e.g., using a 200 mm standard Si {100} wafer as specimen. Consider that the electrolyte needs to contain hydrofluoric acid (HF), often mixed with an organic solvent. This severely limits the choice of materials for the etching cell, electrodes, windows and pumping circuitry, besides causing considerable safety and disposal problems. Looking at a few numbers suffices to turn the so far “simple” etching experiment into a complex undertaking: for typical current densities of (10–100) mA/cm^2^ we need a total current of (3.14–31.4) a flowing at potentials of a few Volts, necessitating heavy-duty contacts and wiring. Bearing in mind that a current density of around 30 mA/cm^2^ is produced by a very good solar cell at “high noon” gives an idea about the dimensions of the light source needed. Using the required kW “light bulbs” for illumination makes a temperature control to at least ± 0.2 ^o^C rather difficult, not to mention space and money concerns. It is thus advisable to employ a matrix of about 4.000 infrared LEDs as a light source, powered by some suitable (and programmable) power supply. Providing an absolutely uniform electrolyte flow across the wafer is not an easy task at these dimensions, and allowing for programmable current, voltage, illumination intensity, and temperature profiles over time, always needed to counter increasing diffusion limitations and voltage losses in ever deeper pores (etching times may be up to 10 hours), calls for specialized hard- and software. Much effort has been extended to meet these requirements; some information concerning these points can be found in [69,70]. Taking everything together, a lab-type large area etcher from ET&TE Etch and Technology GmbH can cost about € 150,000, and a more sophisticated partially automated process station from Semitool will easily go beyond € 1,000,000.

Working with smaller specimens and/or with specimens that do not require dangerous chemicals like HF (most semiconductors besides Si and SiC) or illumination is much easier. The prize to pay is that edge effects can never be completely avoided with small samples, and peculiars of potential measurements, flow control, cell design, ohmic back side contact, and so on, may then cause poor reproducibility and questionable comparisons between results of different groups obtained at nominally similar conditions. Flow conditions, for example, may become critical while etching pores close to some growth mode transition point [14,52,63,65,71] in Si, Ge, or InP, because rather small variations in external parameters might drive the system into one growth mode or the other; this will be illustrated later in this paper.

It is now important to appreciate an essential point in semiconductor pore etching: the system has several degrees of freedom in its external and internal parameters. Omitting the temperature for the time being, we note that we have up to three external variables: current *I*, electrode potential *U*_E_, and (on occasion) illumination intensity *P*.

**Figure 5:**
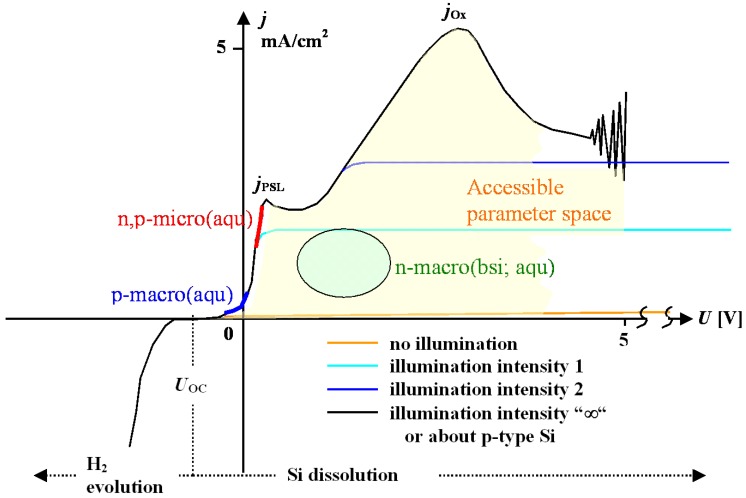
Basic n-Si *I*(*U*) characteristics in about 1 wt% HF aqueous electrolyte with representative numbers. Parameter spaces are indicated. Electropolishing refers to dissolution without pore formation usually accompanied by strong current / voltage oscillations. The two characteristic peaks are denoted *j*_PSL_ and *j*_OX_. For high HF concentration or high doping, pore formation modes change.

The first degree of freedom allows choosing potentiostatic or galvanostatic conditions. Typically galvanostatic conditions (*i.e.*, constant current conditions) are used, because this allows in principle to “drill” the pores under constant dissolution conditions. With illumination as independent parameter, the second degree of freedom comes up. For example, galvanically etching standard n-macro(bsi) pores can be achieved in two basic ways: 1) By using the direct galvanostatic control of the potentiostat/ galvanostat at a fixed illumination intensity *P* - the potential *U*_E_ then must be allowed to adjust to whatever value is needed. 2) By setting both current and potential to fixed values–the illumination intensity *P* then must be allowed to adjust to whatever it takes via a feedback loop. The latter case is typically used in n-Si-macro(aqu, bsi, litho) pore etching, with the added complication that typically *I*, *U*_E_, and the temperature *T* are programmed to follow some function of time. We will not go into details of this (cf. [2] for more information), but just use this example to illustrate that there might be more degrees of (external) freedom than just going galvanostatic or potentiostatic, and that etching state-of-the-art pores is no longer as simple as it might appear on a first glance. This is also reflected in the basic Si *IU* characteristics; Figure 5 gives the paradigmatic example for n-type Si.

**Figure 6:**
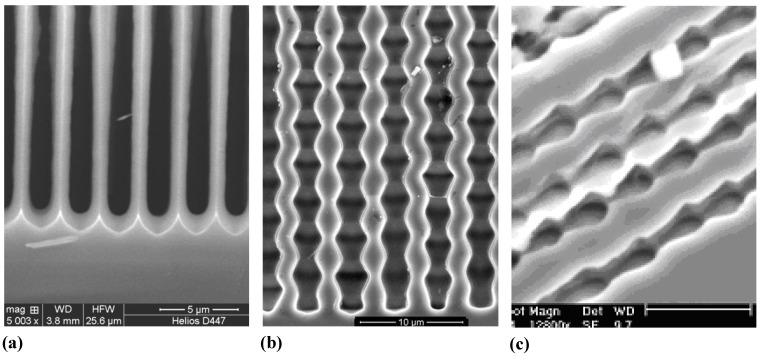
Correlation between pores. (a) p-Si-macro(org) pores. The current through all pores is the same at all times; a self-stabilized pore front results. (b) n-Si-macro(bsi, litho, aqu/viscous) pores. Antiphase oscillations of the current and therefore diameter are observed. (c) n-GaAs-macro pores. Uncorrelated diameter and current oscillations.

Besides the external degrees of freedom the system has at least one internal degree of freedom. Pore etching, by definition, implies that the local lateral current density *j*(*x*,*y*) is larger than the nominal current density *I*/*A* with *A* = sample area, and that it must be a function of the coordinates and possibly of the time. For growing pores the current density needs to be large at the pore tips and small at pore walls and at the surface between pores. In other words, the local pore current density can be an arbitrary function of place and time; *j* = *j*(*x*,*y*,*z*,*t*), with the only restriction that the total current *I* must be constant (for a galvanostatic experiment) at any instance in time. This is trivial and complex at the same time. It is trivial because it simply states that the dissolution is not homogeneous in time and space. It is complex because it necessitates that pores grow “electrically” correlated. If, for example, the current inside one pore decreases, it must increase somewhere else in order to keep the total current constant. If, to give another example, the nominally flowing current density inside some pores (e.g., close to the O-ring) would require a lower potential than for those in the bulk—it can’t be, there is only one potential and it is the same everywhere (neglecting ohmic losses). We now grow some different pore structure at the edge by necessity. Figure 6 illustrates some more subtle consequences of this; more will follow.

The pore structure revealed by looking at a cross-section (after cleaving the sample) is always post-mortem and *ex-situ*; it shows an indirect representation of *j*(*x*,*y*,*z*,*t*). It is indirect because it is not possible to unambiguously assess the development in time of a final pore structure. Did a macropore grow into the depth with the final diameter observed *ex-situ*, or was there some lateral growth, more or less independent of the growth into the depth? In other words: would the pores be thinner if one would look earlier? Do side pores develop later, *i.e.*, nucleate at the pore wall after the pore tip has moved down, or always right at the tip?

*Ex-situ* pictures alone thus cannot reveal the development over time of certain pore shapes and structures; cf. also for example [50,51,52,53,54,65,72,73]. It becomes apparent at this point, that without *in situ* measurement techniques capable of providing some data about what is going on at the pore tip in the depth of the sample, progress in understanding pore formation is severely handicapped.

### 2.2. Current and Dissolution

It is generally assumed and true that anodic currents etch semiconductors. This means that the semiconductor is connected to the positive terminal of the potentiostat/galvanostat (PG), current flow thus involves holes flowing out of the valence band of the semiconductor or electrons flowing into the conduction band of the semiconductor. Both processes occur. In the case of Si, for example, there are good reasons to assume that the arrival of a hole at the Si-electrolyte interface leads to the breaking of one Si-Si bond plus the injection of one electron under certain circumstances (by “ripping off” a second bond). While this dual-carrier process (plus its variants not discussed here) is already sufficient to render the detailed dissolution kinetics fairly complex, the reality is even more complicated, because generally more than one basic dissolution mechanism exists. In the case of Si this is certain—we have direct electrochemical dissolution and dissolution by electrochemical oxide formation, followed by chemical oxide dissolution. Gross reaction equations for the three processes are given below: cf. [11] and most of the books quoted earlier give details.

Si + 6 HF + 1 h^+^ ⇒ [4H^+^ - SiF_6_^2-^] + H_2_ + 1 e^-^ (1)

Si + 2 H_2_O + 4 h^+^ ⇒ SiO_2_ + 4 H^+^ (2)

SiO_2_ + 6 HF ⇒ SiF_6_^2-^ + 2 H^+^ + 2 H_2_O (3)

Equation (1) describes direct dissolution or divalent dissolution with a valence of *n* = 2 (we neglect here some possible complications that could produce n ≠ 2; cf. [74]). The valence of a dissolution process simply gives the number of carriers needed to flow through the external circuit to dissolve one atom of the electrode material, *i.e.*, to remove one Si atom from the crystal in this case. In Si one expects *n* = 4 for basic reasons (four bonds must be broken); smaller values then simply herald “chemical currents” flowing as sketched in Figure 4 with concomitant changes (here H_2_ evolution) of the electrolyte.

### 2.3. First Insight into Pore Formation

Equations (2) and (3) describe the tetravalent dissolution via oxide formation. The valence then is always exactly *n*_ox_= 4. If we now consider the experimentally well established fact that n-Si-macro(bsi, aqu) pore etching only proceeds for *n* ≈ 2.7 [2], we have many of the complexities of pore etching in a nut shell since some unavoidable conclusions follow straight away for Si:

1) Direct dissolution and dissolution by oxide formation must occur “simultaneously” in a mix that produces a valence between 2 and 4.
2) This is, in a strict sense, impossible. One cannot have three different reactions taking place simultaneously, *i.e.*, exactly at the same place at exactly the same time.
3) Holes and electrons are involved in the sum total of the dissolution reactions as given above. Holes, however, are the more important species since even for direct dissolution they are needed to initiate the reaction. This goes a long way to explain pore formation in n-type Si by some mechanism of local hole production. *i.e.*, junction breakdown at high field strength.
4) On the other hand, it also implies that we will run into a problem understanding pore formation in p-type Si.
5) If the three reactions above do not have nearly identical time constants, the slowest one will dominate the reaction kinetics and thus the maximum local current density.

Before we discuss the five points from above in somewhat more detail, a quick look at the dissolution mechanisms in other semiconductors is indicated. It can be a quick look because the message is simple: not much going beyond the contents of the book of Notten, van den Meerakker and Kelly [12] is known with certainty for III-V semiconductors. Rather than going into a prolonged discussion of what might be happening in various circumstances, we prove this claim by relating recent results of the Etcheberry group for InP [75], where detailed investigations into the dissolution mechanisms and the concomitant valence for InP pore formation raised far more questions than were answered. The situation is hardly better in the case of SiC, Ge, or the II-VI semiconductors. Nevertheless, since at least for compound semiconductors rather more than less basic dissolution mechanisms exist in comparison to Si (one could, for example, form In-oxide and/or P-oxide in several variants with InP), it is not too far fetched to assume that more than one gross reaction is occurring or even needed during pore formation in other semiconductors, too.

We therefore restrict the discussion of the five points from above to Si, but keep in mind that some, if not all of the reasoning, applies most likely also to other semiconductors.

The dichotomy between **point 1 and 2** is easily resolved. All one has to assume is that the three processes occur decoupled in time and space on a microscopic scale but with constant averages on a macroscopic scale. In other words, on a small (some nm^2^) area of the electrode at *t* = *t*_0_ only direct dissolution might take place, followed by oxide formation at *t*_1_ that is finished at *t*_2_, and the subsequent dissolution of the oxide until *t*_3_.

There is no logical way around some scenario like this and this has far-reaching consequences. In the time span between *t*_2_ and *t*_3_ no (or very little) current will flow through the small oxide covered area considered. Current flow will start, as soon as the oxide is thin enough (and therefore the field strength sufficient to cause some break-down) at *t*_3_ and stop if a sufficiently thick oxide has been produced again. In other words: the local current “oscillates” in some stochastic manner and there is no such thing as a constant current density, at most it is smoothly varying with time and location (and describable with standard differential equations). In the opinion of the authors this basic “current burst” hypothesis is not only logically unavoidable but proven by the many oscillatory phenomena found in semiconductor electrochemistry including pore etching (see below or [76,77,78,79]).

**Point 3** emphasizes the necessity of holes, and this explains quite simply why pore etching seems to be easier in n-type semiconductors than in p-type ones. If holes are a-plenty, as in p-type materials, the dissolution reaction could occur everywhere and the kinetics would not be easily limited by the hole concentration. Contrariwise, in n-type material the necessary hole concentration must be produced far above equilibrium levels, if the reactions are to proceed with some appreciable “speed”. To give an example: the maximum current that can be carried by equilibrium holes (= minority carriers) in typical 1 Ωcm n-type Si is < 1 µA/cm^2^, while the etching currents typically used are in the 10 mA/cm^2^ region. There are two ways to produce not only a sufficient non-equilibrium concentration of holes, but to induce pore growth at the same time.

- Production of holes by illuminating the back side. Some of these holes will diffuse to the reaction front at the front side and will “automatically” end up at the pore tips (or tips initiated by lithography) because the concomitant bending of the space charge region cannot but focus holes at the tip. This SCR or “Lehmann/Föll” model [66] was (and still is) the basic model for the formation of the n-Si-macro(bsi, aqu, litho) pores. It was proposed to understand what we now would call n-Si-macro(fsi, aqu) pores, and the discovery that the first experiments immediately confirmed the prediction of the SCR model for n-Si-macro(bsi, aqu, litho) pores was seen as sufficient proof for the validity of the model for this mode of pore etching. Meanwhile, however, it has been shown that n-Si-macro(bsi/fsi, aq, litho) pore etching is more complex and not completely explained by the SCR model [11]. The SCR model is also restricted to semiconductors with a very large diffusion length of the minority carriers—in practice to very good single crystals of Si an Ge. However, in the case of Ge, back side illumination does not appear to influence pore formation very much [52], again questioning the general validity of the SCR model.
- Production of holes by electrical breakdown (tunneling or avalanche type) at points of high field strength = tips of pores (breakdown model, cf. [19]). Local hole production by breakdown is certainly a good reason to form pores in n-type semiconductors of all kinds, and there is no doubt that the breakdown model works, cf. for example, [19].

Nevertheless, local hole production by either mechanism cannot explain all pores found in semiconductors. Micropores (= nanopores in colloquial terms) frequently produced in n-type and p-type Si (and recently also found in Ge [30] but not yet conclusively in e.g., III-V’s ( cf. [80]) must have a formation mechanism of their own, the same is true for the many kinds of pores found in p-type Si and p-type Ge but not (yet) in p-type III-V semiconductors.

The observed anisotropy in pore growth (macropores in Si prefer to grow in <100> or <113> direction; in III-V semiconductors usually the <111>B direction is preferred) is also not easily explained by localized hole generation.

**Point 4** focuses this point on pores in p-type semiconductors; at present this means almost exclusively pores in Si and Ge. Holes are aplenty and there are no conceivable gradients in the concentration with a maximum at pore tips that could easily account for pore formation. This is not to be confused with a lack of holes (and electrons) in space charge layers that still may exist around existing pores at the small forward biases typically used for etching, and that may “protect” the pore walls to some extent.

One good reason for pore formation in this case can be found in a general instability of the advancing dissolution front with respect to disturbances with the “right” wavelength. This has been treated in some detail for Si [20,81], and many aspects of macropores in p-type Si emerge form this model (but not necessarily for pores in p-type Ge).

The key word in this connection is “surface passivation”. A pore, by definition, continues to grow in any material under any conditions if more current flows through the tip than through the walls. If in a thought experiment we look at a freshly generated piece of surface, many reactions can take place. Besides typically several dissolution reactions competing with each other as pointed out above, there are also reactions that do not dissolve the material but “simply” cover or functionalize the surface with some particular species. On a freshly etched Si surface, for example, the Si surface atoms initially are fluorine terminated but if one waits long enough, H-termination will be found since it is kinetically most stable. The most stable surface configuration by definition is more difficult to attack than some less stable configuration; H-terminated Si surfaces therefore are “passivated” against further dissolution in a relative way. For InP, “passivation” probably calls for Cl termination [82]; but not too much is known about passivation for other semiconductors. Passivation is a relative term. It does not mean that nothing can happen anymore, only that passivated surfaces are less reactive than un-passivated ones.

Passivation involves a reaction that takes some time and thus comes with a time constant. This time constant does not only depend on the general electrolyte composition, pH, and temperature, but also on the crystallography. For example, {111} planes in Si passivate faster and are more stable than {100} planes [83,84,85]. It is thus not too difficult to conceive scenarios, where a current flowing through some semiconductor surface is originally uniform, *i.e.*, the current density is same at every pixel (*x*, *y*) but then decomposes into passivated areas, and areas that carry the current if the system is unstable with respect to small disturbances. Pores then develop automatically and with some close relation to the crystallography via lattice plane {hkl}dependent passivation kinetics. 

**Point 5** is trivial—up to a point. The point is that not only the dissolution reactions and the passivation kinetics discussed above all come with some specific reaction time constant tied to “chemistry”, including transport up and down the pores, there are also time constants tied to the semiconductor. Again, n-Si-macro(bsi) pore etching is the best case for discussing this but the general argument applies to all semiconductors. Backside illumination generates holes close to the backside, and these holes must diffuse to the solid-liquid interface at the pore tip. It takes some time *t*_diff_ = *l*^2^/*D* (*l* = distance between back side and pore tips, *D* = diffusion coefficient of the holes) to do this, and *t*_diff_ must be smaller than the bulk recombination life time *τ*_R_, if a substantial fraction of the photo generated holes is to reach the pore tips. Some of the holes traveling all the distance may be consumed or “processed” at the pore tips, some may end up somewhere else. Interface recombination velocities *S* that are different at pore tips or between pores, will describe this effect at the (virtual) interface defined by the plane containing all pore tips. We will come back to this later in Section 4 and only note that the transport of holes from the backside to the pore tips contains several semiconductor related time constants.

Which one of the many time constants discussed so far limits the reaction kinetics as a function of time? Not an easy question to answer, but some points to that will be made in Section 4.

From what has been outlined above it follows self-evidently that *in situ* measurements are definitely needed. The catch is that it is simply impossible at present to “look” down one long, narrow, and not necessarily straight pore to “see” what is happening at the growing pore tip. These leaves only to “look” at all the pore tips as an ensemble somehow. What one then “sees” is some average of what is going on in the individual pores all over the sample, and conclusions become difficult if the etching is not rather uniform. The only method for “looking” into deep pores that has been reported so far is multi-mode *in situ* FFT impedance spectroscopy [28] and we will come back to that in Section 4. Looking at very shallow pores or “surface” pores as in Figure 1(i) or as reported in [86] is easier but rather limited. Some infrared spectroscopy [87,88,89,90,91] or atomic force microscopy (AFM) investigations [92,93] did produce significant results under those conditions, too.

**Figure 7:**
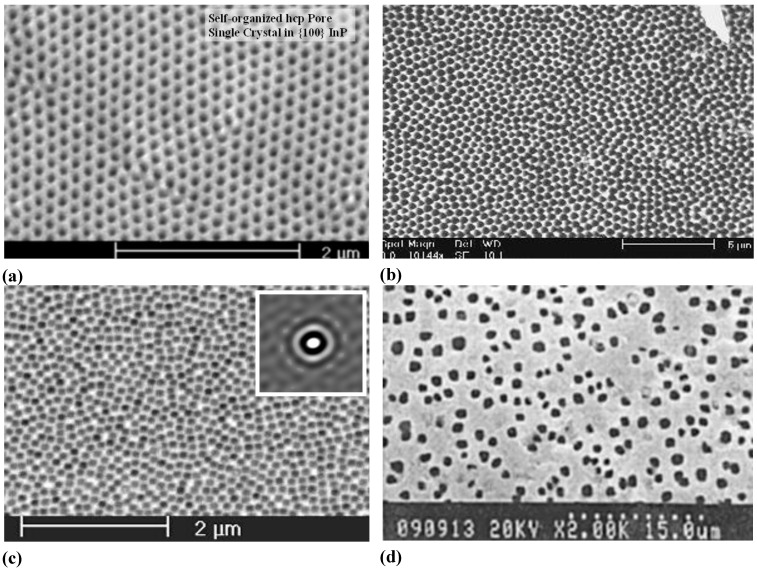
(a) Self-organized single pore crystal in InP demonstrating pronounced current oscillations in space; n-InP-macro(curro) pores. (b) Pore arrangement in Si with pronounced ordering demonstrating damped current oscillations in space. n-Si-macro(org, breakdown) pores. (c) Frustrated pore arrangement with no ordering on the nearest neighbor level but on the second nearest neighbor level. n-Si-macro(org, breakdown) pores. (d) Random arrangement of pores in Si. n-Si-macro(bsi, aqu, random) pores.

Let’s take yet another approach to pore formation in semiconductors that leads over to the next section. We start by looking at Figure 7 where some specific pore arrangements are shown. In Figure 7(a) a nearly perfect self-organized hexagonal pore crystal in InP has formed while in Figure 7(e) a rather random arrangement of pores in Si is shown. The point is that at least Figure 7(a) can be interpreted as a self-organized current oscillation in space. The local current must be large wherever a pore is situated and small in between the pores; and there is an obvious periodicity. The pore arrangement shown in Figure 7(b) with clearly visible short range order then shows a damped current oscillation in space, and so does Figure 7(c) with a “frustrated“ pore arrangement in Si (correlations only to second nearest neighbors; see Fourier transform in the inset and [24]).

Since self-induced current oscillations in time are observed quite frequently in semiconductor electrochemistry (cf. the latest reviews [94,95,96,97,98,99,100,101,102,103,104,105]), the question to be asked is if there is some connection between these two modes of self-organization? The authors are convinced that this is indeed the case and that one of the deeper principles of pore formation in semiconductors is right at the core of both phenomena. This common denominator is the “current burst model” of the Kiel group that will be dealt with in Section 3.4.

## 3. Some General Aspects of Pores in Semiconductors

### 3.1. Major Differences and Common Features

**Figure 8:**
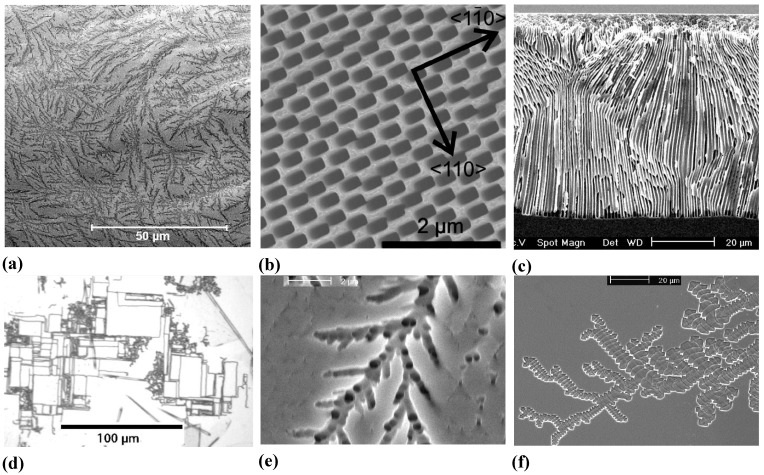
(a) Three-dimensionally distributed fractal pores in ZnSe (cf. [106]); cross-section. (b) Rectangular pores in GaP; top view [29] (with kind permission from Electrochemical and Solid-State Letters). (c) Pore bundle oscillations in InP; cross-section [24]. (d) Mixture of crystallographic and fractal surface pores in Si [107]. (e) Heavily branched pores in Si (n-Si-macro(bsi, org, 111), cf. [108,109]. (f) Surface pores in Si obtained with an ionic liquid (1-butyl-3-methylimidazolium tetrafluoroborate) as electrolyte.

Figure 1, Figure 2 and Figure 3 and many of the following figures prove unambiguously that there are major differences between pores in semiconductors, indeed. Figure 8 serves to demonstrate that there is even more variety than shown here so far [Figure 8(f) has not been published before].

Here we will not dwell on the obvious differences but enumerate major and not always obvious common aspects.

#### Major pore growth direction

**Figure 9:**
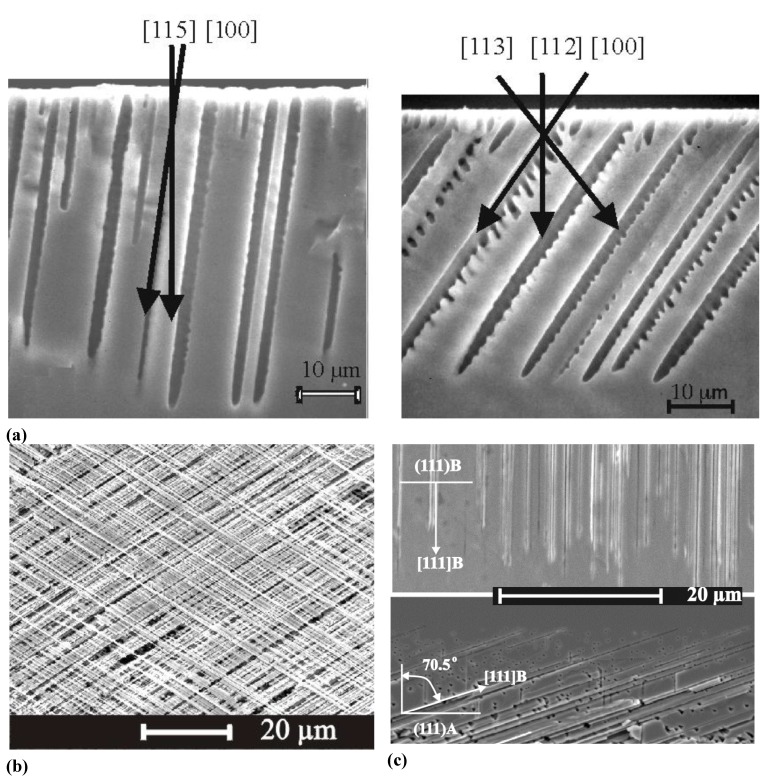

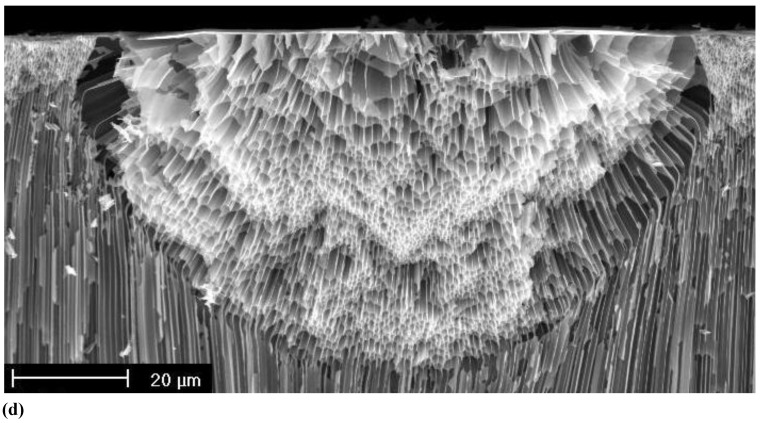
(a) n-Si-macro(aqu, random, crysto) pores in specimens with different surface orientations. (b) n-GaAs-macro(HCl, random, {100} crysto) pores. (c) n-GaAs-macro(HCl, random, {111} crysto) pores growing either from a {111}B or {111}A surface. (d) n-InP-macro(HCl, random, {100} curro) pores with a peculiar “hedgehog” pattern resulting from an initial nucleation at just one point.

Most pores can be classified as either “crystallographic pores” (“crysto” for short) or “current line pores” (“curro” for short) [14]. The pore axis of crysto pores is a crystallographic direction; the pore axis of curro pores follows the flow lines of the current, which are perpendicular to the equipotential surfaces. Figure 9 shows paradigmatic examples. In Figure 9(a) it can be seen that the n-macro(bsi, aqu, random) pores follow the <100> direction even for samples with a surface orientation some degrees off from the usual {100} (left hand side), but switch to <113> if the deviation becomes too large (right hand side); for details see [64,110,111]. 

Figure 9(b) and (d) show pores in GaAs [14,112]. In Figure 9(b) two sets of pores following the two available <111>B directions are seen on the {110} cleavage plane of a {100} oriented sample; in Figure 9(d) the pores were grown from a {111}B surface and from a {111}A surface as indicated. Since only the <111>B direction, *i.e.*, the direction pointing from P to In, is a pore growth direction, the resulting pore structures are completely different.

Figure 9(d) shows a “hedgehog” curro pore cluster in n-InP that turns into more regular downward curro pores eventually. This is easy to understand. Current flow, for some accidental nucleation reason, initially could only occur in a small area in the center of the “hedgehog” (cf. also [113] for similar but controlled pore structures). The equipotential surfaces in the always-present space charge region then are semi spheres for some time, and the current flow lines produce the hedgehog appearance. The picture also illustrates an important point not made so far that is valid for most pores: what one pore does all the others must do (up to a point).

One “spike” of the “hedgehog”, for example, cannot change its direction to “downward” for whatever reason since that would mean that it now must intersect the pores that keep growing in their old directions. Pore intersections are generally not allowed (cf. [58] for details and exceptions). This is a second and so far rarely appreciated principle that couples pores geometrically (the first one provided electric coupling via constant current in time and constant potential in space conditions, see Section 2.1).

Neglecting a few unclear cases (cf. [114] and Figure 12(c)] logic dictates that pores are either crysto or curro. However, what pore type is encountered cannot always be inferred with certainty from post-mortem pictures. Are the beautiful Si-p-macro(aqu+ethanol, litho) pores etched in the group of Bergstrom (see e.g., [115]) crysto or curro pores? As long as the preferred <100> crysto growth direction is identical to the current flow direction (usually perpendicular to the surface), there is no way of telling by just scrutinizing SEM pictures. The example is virulent: in [11] it was conjectured that these pores should be of the curro type, in contrast to the p-Si-macro(org = DMF, DMSO, DMA,… random) pores mostly investigated so far [37,38,116,20,117] and (implicitly) assumed to be of the crysto type; for an exception see [118].

### 3.2. Self Organization and Pore Growth Mode Transitions

As a rule, semiconductor electrochemistry displays many self-organization features. Some are glaringly obvious [e.g., the formation of pore single crystals as a result of self-organization in space; cf. [14,119] and Figure 7(a)], others are not that clearly recognized as a result of self-organization (e.g., the straight pore tip front for many macropores). Pronounced current or voltage oscillations of the Si electrode under potentiostatic or galvanostatic conditions provide perhaps the best-known example for this topic, cf. [94,95,96,97,98,99,100,101,102,103,104,105] for recent reviews. While these oscillations occur in the electropolishing regime, *i.e.*, without pore formation, voltage or current oscillations are also frequently observed during pore growth, cf. [14,24,65,73,119] and Figure 7(a). These oscillations are clearly an expression of self-organization or pattern formation of some quantity in time. As will become clear from what follows, this needs an interaction of the oscillating entities in space.

**Figure 10:**
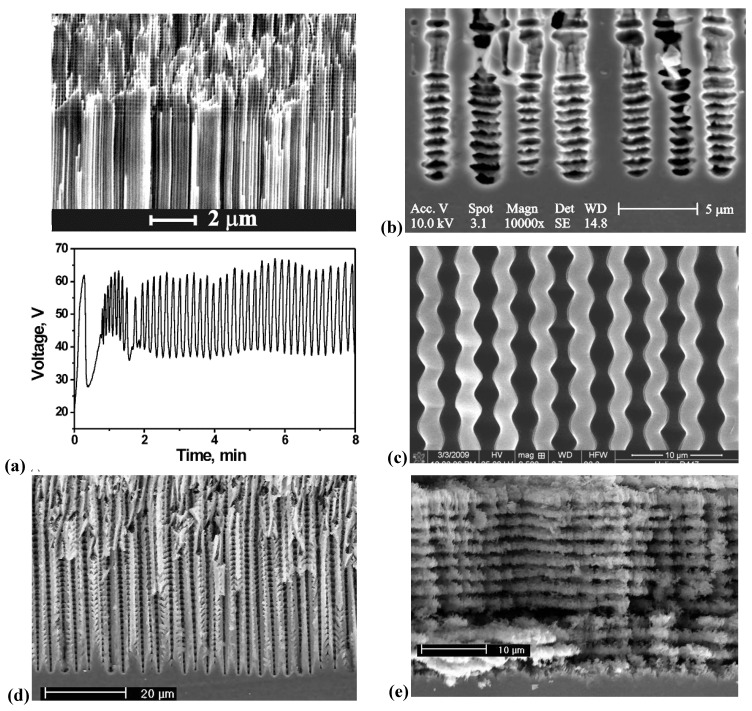
Correlated pore diameter oscillations. (a) Curro pores in InP with self induced pore diameter oscillations in the upper half, accompanied by strong voltage oscillations. (b) Curro pores in InP with self induced pore diameter oscillations in the final stage of pore growth. (c) n-Si-macro(litho, bsi aqu + viscous) pores with antiphase diameter oscillations. (d) Curro pores in Si with pseudo-oscillations. (e) Curro pores in Si with strong correlated pore diameter oscillations bordering on periodic cavity formation producing a “layer cake” structure (see text and [24] for details).

Looking at Figure 10(a) the simple (and so far only) explanation assumes that the current inside any of the pores always tends to oscillate in time. There is no external indication of this as long as the phases of these oscillations are randomly distributed—the total current is the sum of the pore currents and then averages to a rather constant value suiting a constant potential. The assumed current oscillations in a single pore are already an effect of some self-organization, as in the case of the Si current oscillation mentioned above, but will not be identifiable in the external parameters. This should not be surprising at this point since in Section 2.3 good reasons for locally occurring current “oscillations” have already been presented.

Interactions between pores in space that destroy the random phase situation and induce a common phase change the situation completely. The total current could not be constant any more for a given potential and for galvanostatic conditions as shown in Figure 10a), the galvanostat must now counteract this by adjusting the voltage accordingly. This unavoidably produces the voltage oscillations observed. If the phases of the current are different by 180^0^ between neighboring pores (“anti-phase synchronization”) the total current can still be constant but the peculiar anti-phase diameter oscillation as shown in the Figure 10(c) and Figure 6(b) results. Note that this leads by necessity to a frustrated structure akin to the one shown in Figure 7(c); cf. [24,65] for details. 

**Figure 11:**
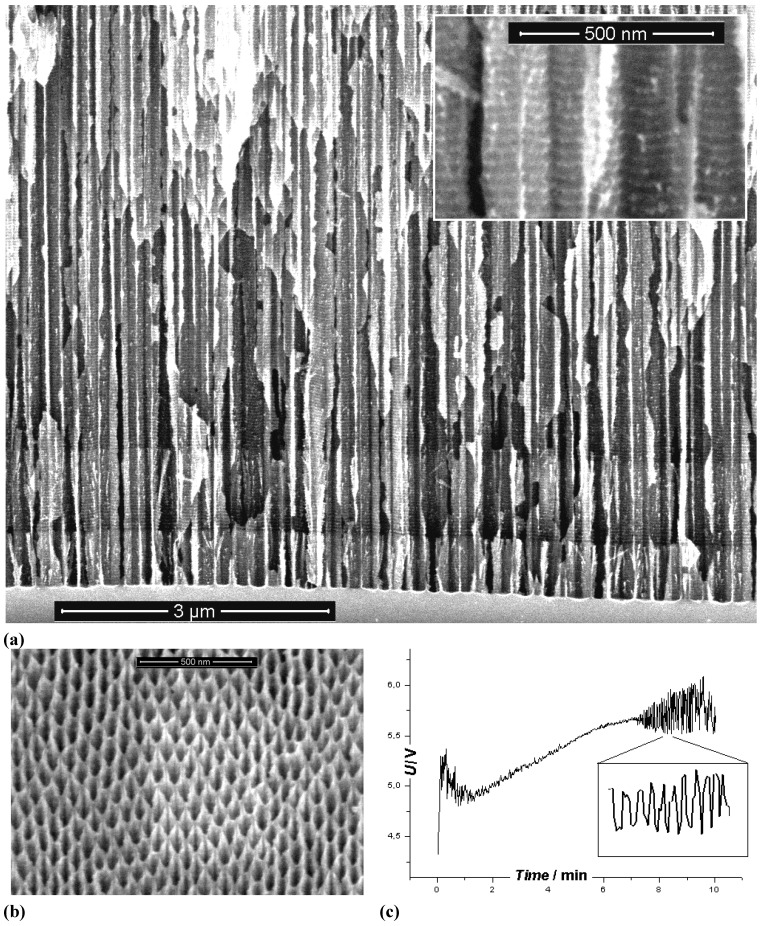
(a) Cross-section of synchronized large-scale diameter oscillations in n^+^-Si-macro/meso(breakdown, random, org) pores; the inset shows details. (b) Top-view (strongly inclined), showing the concomitant strong tendency towards pore crystal formation. (c) External voltage *vs.* time; strong oscillations are observed. The inset shows details.

Generalizing this principle a bit, one can expect macroscopic oscillatory behavior in time (and simultaneously in space, as evidenced by the diameter oscillations shown in Figure 10), if local oscillators become synchronized to some degree. This explanation also holds for similar phenomena observed e.g., in GaP [57,120], GaAs [39,121], as well as for very recent observations concerning n^+^-Si-macro/meso(breakdown, random, org) pores and shown here for the first time in Figure 11. The 65 µm deep pores with diameters in the 200 nm range were galvanostatically etched with a “fast” technique (cf. [109]) in about 10 min. The electrolyte consisted of acetonitrile and HF (2:1). The term “macro/meso” emphasized that those pores are right at the definition edge: the diameter formally belongs to the “macro” regime, but the pore wall thickness meets “meso” criteria. In the general literature, pores like that are usually referred to as “meso”. The resulting structures are not only useful for thermoelectric applications as discussed in Section 5, but show synchronized in-phase diameter oscillations reminiscent of the InP case over large areas of the specimen. The predicted concomitant voltage oscillations were observed as well [Figure 11(c)], as was a strong tendency to form a pore crystal, [Figure 11(b)]. A tendency for some self-organized ordering in pore structures of this kind was also observed by [122].

Generalizing even more produces the following rule.

Macroscopic oscillations (or pattern formation) in time need some interaction in space, and macroscopic oscillations (or pattern formation) in space need some interaction in time. We will come back to this later.

To be sure, not all oscillations observed in semiconductor electrochemistry, or elsewhere, fall under these rules; Figure 10(d) might be such a case. What can be seen are n-Si(random. {111}, aqu. breakdown) curro pores that actually consist of staggered “tripods”, because the pores try to grow in the three equivalent <113> directions, but come to an early stop upon encountering the neighboring pores, and then the process repeats itself. If that is a case of self-organization in the sense as given above is anybodies guess at present.

**Figure 12:**
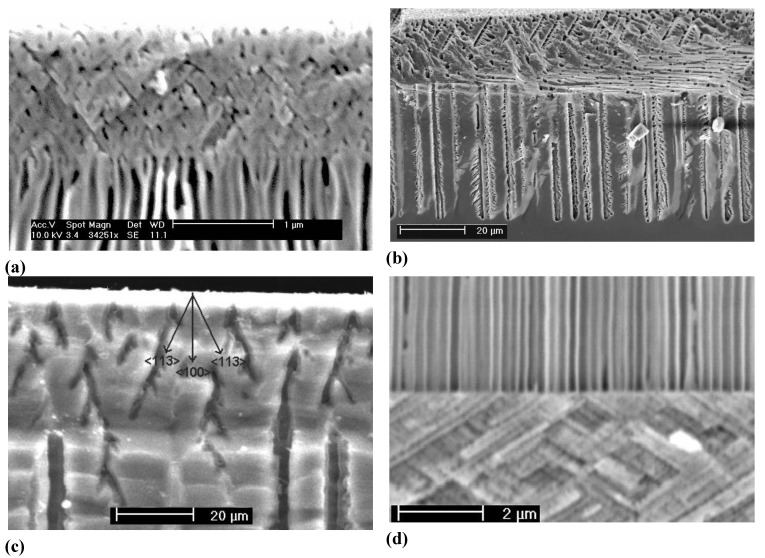
Growth mode transitions of the crysto—curro type. (a) Branching crysto pores in {100} n-type InP with a self-induced growth mode transition to curro pores that will show some ordering (pore crystal formation). (b) Similar phenomena in n-type {111} Si. (c) New finding in {100} n-type Si showing a growth mode transition reminiscent of the {100} InP case. (d) Growth mode transitions from curro to crysto in InP induced by reducing the external current. For details see [14,61,65,71].

Figure 10 shows more than just some “oscillations” in the context of pore formation: we also have what has been termed “growth mode transitions”, cf. [65] for details. The term “phase change” is also found in the literature on occasion [123] but a bit misleading since it always describes thermodynamic equilibrium, not applicable for pore growth. In Figure 10(a) there is a clear transition from a growth mode with synchronized diameter oscillations to one without, Figure 10(b) shows essentially the opposite (transition to stable oscillations) at high magnification. The term “self-organization might also be applied to growth mode transitions, this becomes self-evident in Figure 12.

Figure 12(a) and Figure 12(d) show that a specific growth mode transition may either occur self-induced or externally induced. This is not surprising, because self-induced transitions are triggered as soon as some (gradual) change in parameters reaches a critical value. This explains why growth mode transitions are usually only observed if the system had sufficient time (= large pore depths) to suffer substantial parameter changes, or if it was started at extreme conditions [e.g., a large current density close to the maximum of what can be processed at a pore tip at full potential, *i.e.*, only for not-too-deep pores as in Figure 12(a)–c)].

**Figure 13:**
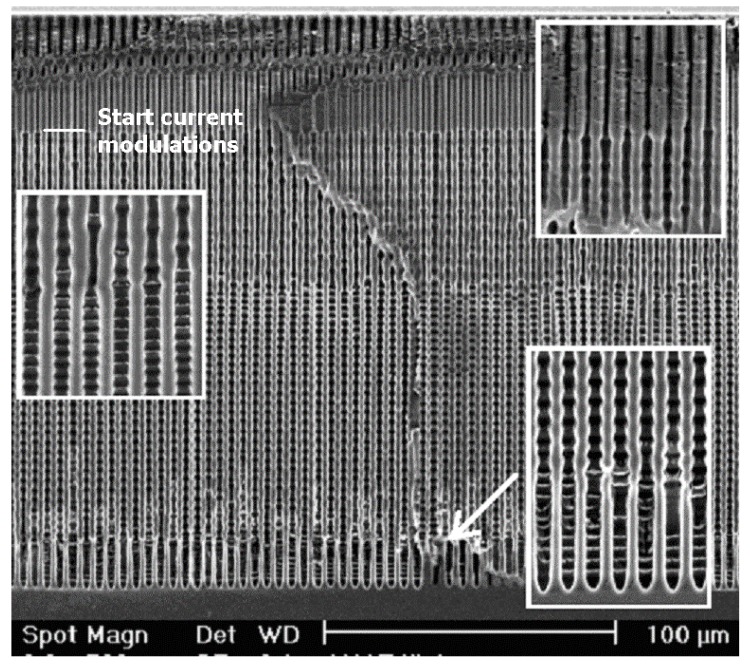
n-Si-macro(aqu+HAc, litho, bsi) pores, growing with a 50% modulation of the current, starting at a depth of about 40 µm. The insets illustrate the growth mode changes encountered. For details refer to [65].

There are many more examples of growth mode transitions; here we will only look at a few of the more spectacular ones in Figure 13. Shown are so-called “fast” n-Si-macro(aqu+HAc, litho, bsi) pores, grown far faster than hitherto possible in a modified viscous electrolyte containing acetic acid (HAc) [for viscosity carboxymethylcellulose sodium salt (CMC) was added] [65,124]. After the pore had reached a depth of about 40 µm, the current was modulated by switching it periodically (*t* = 2 min) between the nominal value and ½ of that value; for details see [65]. Besides the induced growth mode change around 40 µm depth, two abrupt self-induced growth mode changes are visible, where the pore diameter modulations tied to the current modulation suddenly change their “wavelength” and thus the growth speed.

**Figure 14:**
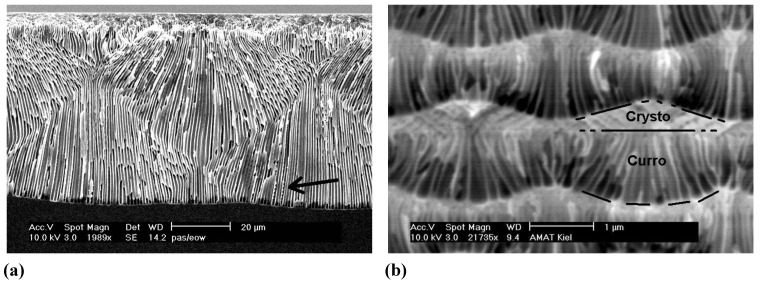
Self-induced pore-bundle diameter oscillations. (a) Curro pores in {100} n-type InP; wavelength ≈ 80 µm. (b) Externally induced periodic switch between crysto and curro pores with self-induced pore bundle oscillations in parts of the depth. Wavelength ≈ 2 µm.

We will make no attempt to discuss what is going on here (it is not fully clear at present anyway, cf. [24,65]), but just point out again that one can look at ex-mortem pictures like the one in Figure 13 (or related ones) for any amount of time without being able to deduce what was going on. The same is true for the pore structures shown in Figure 14. It is becoming obvious once more, that progress in understanding pore formation in semiconductors needs some *in situ* assessment of relevant parameters. Figure 14 finally shows some unique cases of pore-bundle oscillations in space, so far only observed in InP. The diameters of single pores oscillates in an unsynchronized manner [the arrow in Figure 14(a) points to some faintly visible cases], but the diameter of a whole bundle of pores oscillates quite strongly and in some anti-phase synchronization reminiscent of the Si case shown in Figure 10c) for single macropores. In Figure 14(a) we have completely self-induced pore bundle oscillations of curro pores, in Figure 14(b) a periodic crysto-curro-crysto transition was externally induced in addition, but this does not normally induce pore bundle oscillations [14,125].

The point of this sub-section has now been made: self organization phenomena (including pore growth mode transitions) are not only rather common in semiconductor pore etching, but show close similarities between completely different cases. This proves that some “meta” principle must be involved in pore formation that transcends the detailed and specific chemistry of dissolution.

### 3.3. Pore Geometry

**Figure 15:**
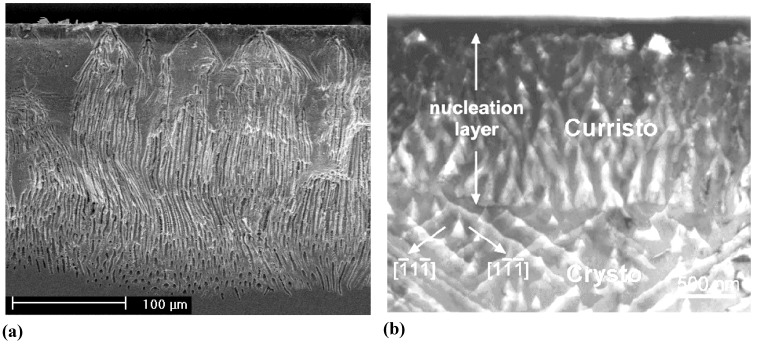
(a) SEM cross-section of very inhomogeneously nucleated n-Si-macro({111}, org, dark, breakdown) pores [109]. (b) Transmission electron microscopy (TEM) cross-section of pore nucleation in InP [126]. There is a thin nucleation layer of mixed pores (“curristos”; cf. [114]) before regular crysto pores are formed.

The geometry of pores, meaning essentially their average diameter and the average distance between pores, is either determined by structured seeding or nucleation (“litho”), or determines itself via “random” nucleation, often followed by some rearrangement of the initially nucleated pore structure. In the first case the pores may or may not nucleate at the sites provided - the size of the related process window seems to be rather large for macropores in Si and rather small for most everything else [49,50,64]. The decisive quantity seems to be to what extent the geometry of the pattern provided by lithography is commensurate with the pattern the pores aspire to achieve by self-organization. Many of the pictures here and elsewhere show rather uniform structures with rather small variances in the geometric parameters if not almost perfect pore crystals ([Figure 7(a), Figure 11(b)]. To be sure, there are many exceptions, *i.e.*, very irregular structures, but that seems to be mostly due to inhomogeneous nucleation and not enough time for a pore structure rearrangement with depth; Figure 15(a) shows an example.

Note that a top view in the case of Figure 15(a) would show a very inhomogeneous pore structure with a geometry completely different from that somewhat deeper in the sample. Note also that in many cases the original surface is no longer present (cf. [127] for a detailed study), *i.e.*, some of the original nucleation layer has been dissolved. We might thus conjecture that in most if not all cases of pore etching, there is a preferred structure with a rather well defined geometry, *i.e.*, strongly preferred pore diameters and distances. In other words, there is some well-defined intrinsic length scale inherent in the pore etching system that is expressed in some specific pore geometry. This has been discussed in some detail in [13], here we just note the most important lengths scales typically encountered:

i) Width *d*_SCR_ of the space charge region (SCR). This is strictly a function of doping (or more generally the majority carrier concentration) and the potential. The range of *d*_SCR_ (for 1 V; multiply by 3.16 for 10 V) scales from 10 µm for low doping of 10^13^ cm^-3^ (corresponding to *ρ* ≈ 250 Ωcm) *via* 100 nm to 10^19^ cm^-3^ (corresponding to *ρ* ≈ 0.005 Ωcm) for n-type Si.

ii) Critical tip radius for electric breakdown (= hole formation) to occur. This is also a function of doping and potential; some details can be found in [19]. There is no simple equation correlating the breakdown field strength *E*_BD_ to the pore radius *r* or more precisely to the radius of curvature of the pore tip, with the resistivity as parameter, because there are at least two different breakdown mechanisms (tunneling and avalanche effect; cf. [128]), and the exact shape of the pore tip will be of importance. From the graphs given in [2] one can deduce the following numbers: for pore tip radii of *r* ≈ 10 nm–20 nm, breakdown occurs for voltages around 1 V for the complete typical range of doping (*N*_D_ ≈ (10^15^–10^18^) cm^-3^); for heavily doped Si (*N*_D_ ≈ 10^18^ cm^-3^) breakdown always occurs at voltages below a few Volts. The breakdown field strength for a given set of parameter might also be direction dependent; cf [128] for the case of GaAs.

iii) Length scales not determined by the semiconductor and given, e.g., by lithography, diffusion in the electrolyte, quantum confinement, or by so far unknown mechanisms. For example, the 80 µm scale inherent in the pore bundle diameter oscillations shown in Figure 14(b) can hardly be found in the semiconductor, but might relate to the angle between the electrolyte flow direction and the <110> crystal direction. The nm length scale found in micropores (and “small” mesopores) relates most likely to quantum confinement as suggested [67,68,129], *i.e.*, to the general effect of “nanowires” etc. that the effective band gap of a semiconductor increases for small dimensions of the crystal.

It is important to note that a growing pore cannot choose its diameter “at will”. Neglecting quantum confinement effects (*i.e.*, micropores), the distance to neighboring pores has to be at least *d*_SCR_ since the SCR is effectively an insulator. For a given general geometrical structure (e.g., by lithography) this imposes also an upper value for the diameter. A lower value is given for many reasons, the simplest ones being that breakdown will occur for too small diameters. At this point we note that pore growth in the “breakdown mode” (pretty much all III-V pores) does not assume dimension where breakdown starts but where it just stops. That happens to be the same practical value, but there is an important difference in visualizing the effect. “Avalanche” breakdown, as the name implies, would produce an arbitrarily large amount of holes and thus large local currents. The concomitant chemical reactions then will always stop the process—either by forming oxide or by increasing the diameter. The response to a sudden current surge at a pore tip because some breakdown event has started (in other words: a current burst was initiated) thus is always that the current is stopped again by the interplay of the various dissolution processes. Some steady state would be conceivable in principle but is highly unlikely, given the intrinsically stochastic and highly non-linear nature of breakdown events (there is no such thing as a straight and sustained bolt of lightning in a thunderstorm or a steady unchanging arc in welding, even so it’s not forbidden).

In between some limits for the pore diameter *d*_Pore_, the pore has an interesting degree of freedom. A certain fraction *I*_Pore_ of the (constant) external current *I* flows through a pore; in the simplest case of *z* identical pores we have *I*_Pore_= *I*/*z*. The current density *j*_Pore_ then is nominally 4*I*_Pore_/ π*d^2^*_Pore_. However, at some differential area d*A* positioned somewhere on the pore tip it can (and actually must) have a quite different value. A pore now has the freedom to choose a certain nominal current density by adjusting its area within the limits given. Moreover, if the current changes, e.g., because the external current is changed, the pore can react by either changing its diameter to keep the current density constant or the growth speed to keep the diameter constant, or by suitable mix of the two. All pores together even have a third option: they may enact a growth mode transition, e.g., from crysto to curro or *vice versa*.

It is not obvious what a pore or the pores are going to do, and what the criteria for the choice will be. Moreover, whatever the pore wants to do must be “checked“ to some extent with its neighbors because, as pointed out above, a single pore is always correlated to its neighbors electrically and geometrically. This coupling becomes most important if the pores want to choose a growth mode transition as the third option.

Most of what was stated above applies to macropores, *i.e.*, pores with diameters > 50 nm. For everything smaller the situation is not so clear. True nanopores so far appear to be restricted to Si (plus one recorded case for Ge [30]). Since it was difficult to generate clear pictures of very small pores with an SEM (pore research rather predates the advent of high-resolution SEMs), reliable detailed geometric data are scarce. Nevertheless, many aspects of meso- and micropores can be included in the general approach pursued here as the next sub-section endeavors to show.

### 3.4. A Closer Look at Self-Organization Issues

It is fair to say that the current or voltage oscillations found with a Si electrode under potentiostatic or galvanostatic conditions, as mentioned in Section 3.2, may be seen as paradigmatic for the topic of semiconductor electrochemistry self-organization. The first quantitative model for these oscillations was the so-called “current burst model” (CBM) of the Kiel group first published in [130,131]. While the CBM is not uncontested [94,95,96,97,98,99,100], it has by now quantitatively explained more experimental observations than its competitors with less basic assumptions [104]. The CBM, in a generalized and more qualitative version, has also been applied to pore formation and yielded a number of insights and several predictions, many of which were proven true in the meantime [132]. The authors are therefore convinced that the CBM provides one of the general principles underlying the common aspects of pore formation although it is not capable of explaining all aspects of pore formation. It will be introduced here very briefly, more detailed recent overviews can be found in [11,24].

**Figure 16:**
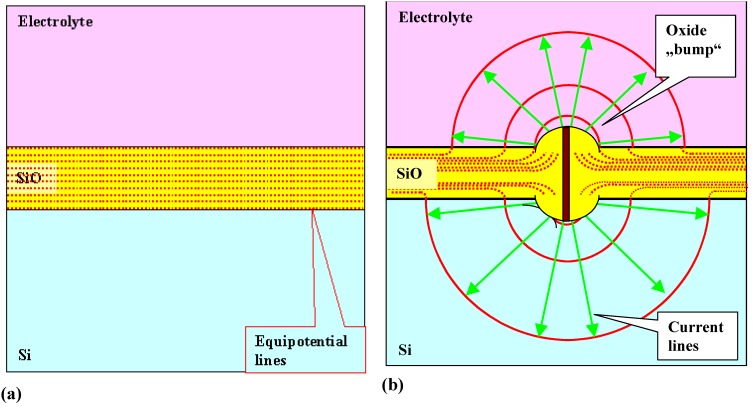
Schematic illustration of a current burst through an oxide layer. (a) Dissolving oxide layer. (b) A current burst occurs with some probability at high field strength = thin oxide, initiating rapid oxide growth and a concomitant redistribution of current and field lines.

For the sake of simplicity let’s look at a p-type Si electrode covered more or less uniformly with oxide in an HF containing electrolyte and at a potential where current flow is not possible at the beginning of the experiment, cf. Figure 16(a) for a schematic drawing of this situation. The applied potential drops almost completely in the insulating oxide, and all that happens in the beginning is that the oxide dissolves chemically. Under such circumstances it is usually assumed without further reasoning that current flow starts as soon as the oxide is dissolved—but that is wrong. The field strength in the oxide = *U*/*d*_ox_ reaches values large enough for electrical breakdown before its thickness *d*_ox_ is zero. As a quite good rule of thumb, the highest field strength the very best dielectrics can bear is about 10 MV/cm or 1 V/nm. Above that ultimate field strength electric breakdown (and current flow) is unavoidable, and for less than top-quality oxides the breakdown field strength can be considerably lower than the number given above. Violent electrical breakdown always has a stochastic component: it never can be predicted with certainty exactly when and where it will occur (witness any thunderstorm). The first ingredient of the CBM then is to assign some probability function for breakdown to occur at some field strength to the oxide barrier of our example (or to any other barrier or “passivating” layer opposing current flow). After a breakdown has occurred, current flows and produces an “oxide bump”, as shown schematically in Figure 16(b). Note that the shape of the oxide bump is unimportant, all that is needed is that it has some lateral extension scaling with the oxide thickness. The field strength in the growing oxide bump decreases and there is now a certain probability assigned to the process (increasing with decreasing field strength) for current flow to stop.

The equipotential planes and the current flow lines will be about as indicated in Figure 16(b) but that doesn’t need to be conjectured, it can be calculated given the conductivities of the Si and the electrolyte.

The two probability functions discussed above are the only basic ingredients of the CBM. Their precise mathematical formulation (cf. e.g., [104,130,131]) follows established procedures for this kind of modeling and contains the only numbers not deducible from first principles. Everything else needed for a quantitative Monte-Carlo implementation of the model can be calculated in principle. The current flow through a Si electrode is now simply given by summing over the current flowing in locally and temporarily active current bursts. Note that in any given pixel (≈ nm^2^) of the electrode, most of the time no current is flowing because it turns out that oxide dissolution is the process with the largest time constant and thus dominates the kinetics.

The decisive issue is that current bursts “automatically” (*i.e.*, without further assumptions) correlate in space (and time). It is less likely, for example, that a new current burst starts close to an old one because the oxide in its neighborhood is thicker than somewhere else. In other words, there is an interaction in space and that does indeed produce “automatically” the observed oscillations of the current (or voltage) in time, cf. [104] for an in-depth review.

Section 3.2 stated “macroscopic oscillations (or pattern formation) in time need some interaction in space”. If we consider pores to be more or less damped oscillations in space, as outlined in Section 2.3 (cf. [24,104]) this would require some interaction in time in terms of a generalized current burst approach.

**Figure 17:**
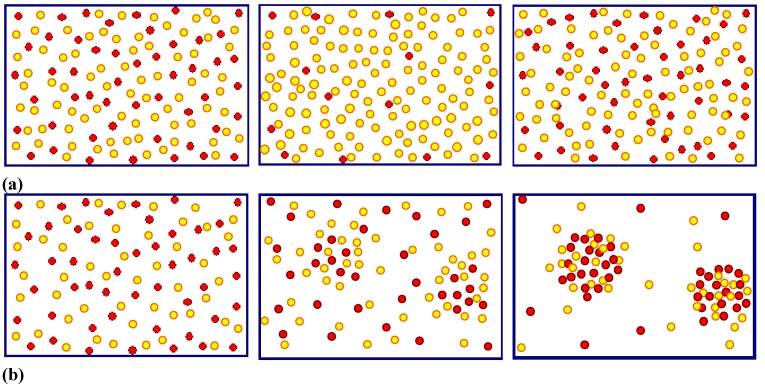
Schematic time sequence. Time increases from left to right, red dots mark active (current bearing) current bursts, yellow circles inactive ones just dissolving oxide bumps. (a) Current oscillations in time. (b) Formation of current oscillations in space or (macro) pore formation.

Some interaction in time between current bursts is exactly what happens if we replace an oxide as passivating layer by anything else that can render current flow locally more difficult, e.g., a completely hydrogen “passivated” part of the Si—electrolyte interface. Let’s consider some area where a current burst stopped some time ago and the oxide formed in the active part of the current burst now dissolves. Even for very thin oxides or no oxide, a new current burst may not nucleate for several reasons. For example, many other nearby areas might be “very busy” and draw all available current at galvanostatic conditions, or no holes are around at that location, because we have n-type Si, or for a number of other easily conceived reasons. The oxide then is completely dissolved after some time and the bare Si surface starts to passivate *via* hydrogen coverage. How long that takes, and how well this passivation protects the Si, depends on the crystallographic orientation, the pH value of the electrolyte, and many other parameters. The consequence for a generalized CBM is simple: the probability function for the breakdown of some passivating layer at time *t* and a given position (*x*,*y*,*z*) on the pore-electrolyte interface is no longer just a function of one parameter but of many. While the various functionalities for a certain case are not too difficult to state, the related three-dimensional modeling task is presently not computable. Nevertheless, some quite specific statements or predictions are possible, cf. [36,37,108,133,134]. Here we just consider two:

1). Under conditions where direct dissolution prevails and the current is relatively large (*i.e.*, in the micropore region of Figure 5), each individual current burst generates a nm-sized pore, “dug” by direct “divalent” dissolution and eventually closed by a small oxide lump. All that is required now to form sponge-like microporous Si is what one could call an “anti-correlation” in time: wherever a current burst has stopped, it is less likely that a new one will nucleate a short time after. This is likely to happen if there is a need to keep many current bursts “burning” all the time, and that is the case if the galvanostatically enforced current is close to a limiting value, here *j*_PSL_ (cf. Figure 5). There is not enough time to wait for a new current burst until the oxide lump produced by the first one is sufficiently dissolved and a new current burst nucleates somewhere else—to the left or right of the oxide lump. The growing nanopore must dig into the Si in a kind of random walk (it cannot get too close to other pores, however), and a sponge-like network of nano-sized pores must result. We have not only “explained ”micropore formation in this way, but also the value of the first current peak at *j*_PSL_: it is simply the maximum current observable if the entire surface is covered with current bursts all the time.
2). Under conditions where direct dissolution prevails and the current is relatively small (*i.e.*, in the macropore region of Figure 5), the situation is reversed. Current bursts correlate positively in time, *i.e.*, it is more likely to nucleate a new one in places where an old one just expired. That appears contradictory to the first case but simply states that the nucleation probability as a function of time first is low (the pore tip is still covered with an oxide bump) but then increases (the oxide becomes thinner or disappears) and then decreases again (the surface starts to passivate with hydrogen). If a positive correlation prevails, the current bursts will start to cluster as shown in Figure 17(b) and macropore formation is inevitable

Much more could be said about the CBM and pore formation but the reader at this point is referred to the numerous articles cited in this regard. It is more illuminating to consider present limits of the CBM. First, it must be mentioned that a fully quantitative version of the CBM so far has only been implemented for small HF concentrations and therefore for small current densities. There are several effects (like electron tunneling through oxides) encountered at high current densities that are not easily dealt with, even so they do not appear to be decisive for what is going on. The same is true for other potentially occurring effects, *i.e.*, stress induced by oxide patches [97]. While it might be safe to some content that some current burst like dynamics may always exist if there are several competing reactions at the interface, this has not proven beyond doubt for all possible combinations of a semiconductor and an electrolyte. Moreover, the CBM by its nature does not directly contain length scales tied to instabilities of an advancing dissolution front [104] or the space charge region dimension.

On the other hand, variants of the CBM have been used successfully (and to a large extent quantitatively) to model findings dealing with pure anodic oxidation of Si in various organic electrolytes with bearing on pore formation in those electrolytes plus HF [135], and for the formation of crysto pores in InP and GaAs (see [136]).

### 3.5. A Closer Look at Growing Deep Pores

All things considered, the CBM might be seen as supplying the “stochastic” ingredient necessary to understand pore growth in general and many of the self-organization features encountered. That is not sufficient, however, for understanding all the effects observed. As soon as one tries to grow very deep pores, or tries to grow pores very fast (or both), complex general phenomena are encountered that are not (easily) understood with the CBM alone. The growth mode transitions elucidated in Section 3.2 (and mostly found more recently while attempting the feats listed above) are a case in question, as is the inevitably encountered complete loss of pore growth at some limiting depth (often accompanied by a lift-off of the porous layer).

This needs at the outset a closer look at the more gradual changes occurring smoothly (and not stochastic) in time. This includes the actual potential at a pore tip deep in the substrate together with the local concentration of various chemical species and possibly the hole supply on the semiconductor side. While the potential *V*_tip_ might occur to be the most important parameter that cannot but decrease according to:

(4) $$V_{tip}=V_{0}-j_{\text{pore}}\rho l$$

with *ρ* = specific resistivity of the electrolyte, *l* = length of the pore, and *j*_pore_ = nominal current density inside the pore, this is actually not really the case.

This shall be illustrated by looking at a special task: the very fast growth of very deep n-Si-macro(bsi, aqu. litho) pores. The problem is easily stated: fast growth needs high current densities, and thus such high HF concentrations. Experiments performed with this in mind invariably found, that the higher the initial growth speed, the shorter the maximum depth achievable. The nominal ohmic losses inside a pore [the *j*_pore_*ρl* term in Equation (4)], or the decreasing HF concentration down the pore, and so on, were thought to be responsible for this behavior. However, these problems could easily be remedied to some extent: just increase the potential and the HF concentration as a function of time, and the conditions at the pore tip should not change all that much. Unfortunately, taking measures like this do not seem to work. What does work to some small extent is to decrease the temperature, or to increase the viscosity during the 10 hrs or so of etching.

Both working measures are counterintuitive because they decrease the diffusivity and thus make transport in and out of the pore rather more difficult. However, pore growth most likely does not terminate because it becomes too difficult to transport molecules in and out of long pores. This is illustrated by the rather uninhibited growth of micropores down to large depths, where the reagents must diffuse up and down a very narrow and very long (because tortuously winded) pore, or the rather fast growth of so-called “metal assisted” pores [137]. The reason is rather that some precarious balance regulating vertical (what one wants) and lateral (what one doesn’t want) pore growth must be maintained. A quick look at the particle current flowing into and out of a pore gives some impression of that. First of all, the net electrical current density carried by the ions of the electrolyte must be the same at any depth *z* of a pore, as long as no lateral pore wall currents are flowing. For any charged species the driving forces for that current are the concentration gradients and the electrical field (and both are correlated by the Nernst equation). In addition, the several reactions leading to dissolution may require uncharged species [e.g., HF in the case of Si, cf. Equation (3)], which are supplied to the pore tip by pure diffusion, driven only by a concentration gradient. The pore walls need to be passivated by hydrogen, needing some transport, and so on. Calculating the balance of all these particle currents (including reactions that may take place on the way down the pore, e.g., dissociation of HF) is beyond present capabilities although some valiant attempts have been made [138,139] It can be appreciated, however, that a small increase of the applied potential with time in order to counter ohmic losses, while indeed helping the pore tip to process the available holes by dissolving Si, might also cause an increase of the (small) “leakage” current density at the pore walls. The total current through the pore walls, however, is the pore wall area times the current density and a small increase of the latter by increasing the voltage might simply not leave enough current for the tip because the total current per pore is about constant.

How complex this “chemical” part of a general pore growth model can be is illustrated by the still amazing fact that the addition of some acetic acid to the standard 5% HF based aqueous electrolyte is detrimental for growing good n-Si-macro(bsi, aqu, litho) pores, while it enables quick and deep growth of those pores at 10% and 15% HF concentration [140]. Equally amazing is the often very helpful effect of making the electrolyte more viscous [124,141].

There are some in-depth speculations and reasoning about the what’s and why’s behind these and other “chemical” influences on general issues of pore formation in semiconductors, but the time is not yet ripe for summarizing principles. We rather use the information given so far to emphasize and lead over to the next section: *in situ* measurements during pore growth.

## 4. Multi-Mode-*In-Situ* FFT Impedance Spectroscopy During Pore Etching

### 4.1. General Technique

The need of *in situ* measurements during deep pore etching in semiconductors has been emphasized several times in the preceding sections. There is, however, no obvious monitoring technique out of the long list of possible methods (cf. e.g., the overview of Chazalviel [142]), because it is not so simple to “look” down a very long and possibly crooked pore at the interface Si/electrolyte, where things happen. The only method that can make some claim to *in situ* monitoring is dual-mode FFT impedance spectroscopy (IS), as recently published in [28].

Impedance spectroscopy is a well-established technique in electrochemistry and well documented in several standard textbooks and innumerable articles - but no impedance measurements have been attempted during (macro) pore etching, if we discount [143,144,145,146,147,148], where no deep pores were present. The reasons for this lack of data resides in a large number of “small” problems, that taken together rendered meaningful measurements rather difficult.

Classical impedance spectroscopy is a “black box” method that monitors the response of an electrical system output to small disturbances at its (electrical) input in the form of a *A*’·sin(*ωt*) modulation (*A*’ = (small) amplitude either of the current or the voltage, *ω* = circle frequency of the modulations). Linearity is assumed, *i.e.*, the output can always be written as *A*’’·sin(*ωt* + *ϕ*). The measured quantities then are output amplitude *A*’’ and phase shift *ϕ* as a function of the input amplitude *A*’ and the circle frequency *ω*. Small amplitudes are not only necessary to ensure linear response, but also in order not to disturb the system (cf. the response to large amplitudes in Figure 13 or the growth mode transitions induced by a “modulation” of the current in Figure 12(d).

We first discuss the application of the technique to n-macro(aqu, bsi) pores, and then present a very recent case study in InP and GaAs.

Impedance spectroscopy during pore growth is easy in principle, but not in practice. First of all, the response comes from whatever happens inside of typically a few millions of pores. If the etching process is not very uniform, no meaningful conclusions might be drawn from data that then represent some unclear averages over millions of different pore tips, pore wall properties, and regions around the O-ring, with often quite different structures. This problem can be overcome to some extent with carefully optimized etching conditions.

Second, measuring sequentially for all the required many frequencies may simply take too long—the pore parameters, e.g., its depth, may have changed too much during the measurement. Modulating the input with all the desired frequencies simultaneously, plus performing a (fast) Fourier Transform of the output signal (FFT) solves this problem. However, because the total amplitude of a signal containing many frequencies now is larger, linear response demands smaller individual amplitudes, invariably leading to signal / noise problems. Nevertheless, FFT impedance spectroscopy is a must for fast *in situ* pore growth monitoring.

Third, classical impedance spectroscopy (abbreviated *IV*–IS in what follows) modulates the voltage (or current) and monitors the current (or voltage), and then obtains a proper measured electrical impedance *Z*_exp_ = d*V*/d*I* with the unit Ohm (Ω). Even if good data (= not too noisy and without drift) are recorded, it is not directly clear what those data mean. Without a quantitative model, *i.e.*, an equation for *I*(*ω*,*V*) and thus a theoretical impedance *Z*_theo_ = d*V*/d*I* that contains the primary parameters of interest against which the measured data can be fitted, not too much can be learned off-hand about what is going on inside the pores. We are now caught in a catch-22 situation: a model for *IV*–IS essentially needs a theory of the *IV* characteristics, and such a theory or model does not yet exist. This necessitates the use of “equivalent circuit” models with all the concomitant problems, e.g., the uniqueness and suitability of the circuit chosen [149,150].

Major progress with respect to monitoring pore formation comes from a new mode of IS: modulate the intensity *P*(*t*) of the back side illumination and record the response in the current. As it turns out, this „pseudo” or photo impedance *Z*_bsi_ = d*P*/d*I* (unit is not Ohm) can be derived theoretically in great detail for n-Si-macro(bsi) pores. This mode will be called bsi-IS in what follows. Of course, one could also use modulated front side illumination wherever applicable [13]; then we have fsi-IS that turns out to be extremely useful for solar cell R&D, cf. [28,151,152] for a full discussion of both modes of photo impedance. Using more than one mode finally establishes the dual-mode FFT IS technique that will be introduced for *in situ* monitoring of n-macro(aqu, bsi) pores here. 

It can be appreciated now, why it took the Kiel group many years to produce meaningful IS measurements during pore growth. Some dedicated and highly specialized hardware had to be conceived, built, and tested first.

During an etching experiment that may take several hours, first an *IV*–IS is taken during one second, followed by one second of “rest” for the system, then, a bsi-IS is taken for another second. After another second of rest the process is repeated. The spectra emerging every two seconds are recorded and matched against the theoretical equations given below. This allows to extract a number (around 10) of relevant parameters that are then displayed on-line.

**Figure 18:**
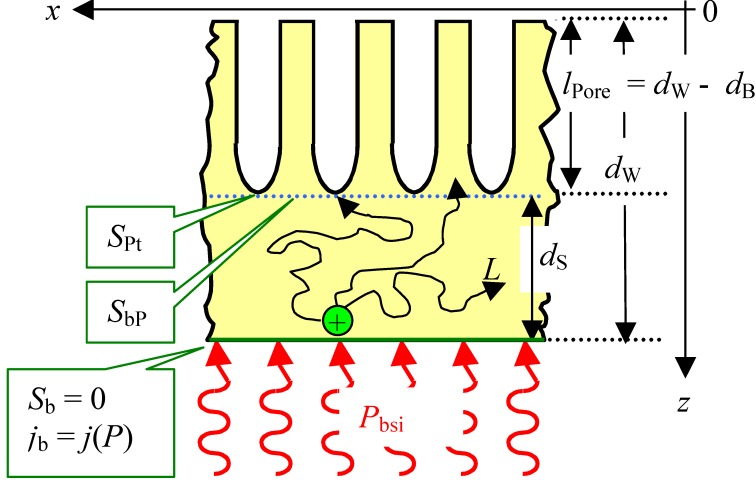
Geometry of n-Si-macro(bsi) etching and some quantities needed for bsi-IS. See the text for details.

First, a fully quantitative model of the bsi-IS mode will be introduced. There are several indispensable parameters that must be taken into account. Besides the geometry, with *d*_W_ = thickness of wafer or specimen, *l*_Pore_ = length of pore and the (modulated) illumination intensity *P*_bsi_, one needs to account for all the sinks encountered by the holes produced by the illumination. Neglecting the effects of the finite penetration depths of the illumination, and recombination of the holes right at the back side (*i.e.*, a back side surface recombination velocity *S*_b_ = 0 cm/s is assumed), for the sake of simplicity (those effects are contained in the software evaluating of the data, though), there are three sinks for holes: i) recombination in the bulk of the Si. This is described by the diffusion length *L* = (*Dτ*)^1/2^ with *D* = diffusion constant of holes and *τ* = bulk life time; ii) the tip of pores, where the holes are converted into an ionic current and trigger the dissolution of Si according to Equation (1); and iii) holes ending up between the pores, essentially producing leakage current (dissolving pore walls). The latter two mechanisms translate into a boundary condition at the plane, defined by all pore tips for the recombination velocities *S*_Pt_ at the pore tips, and *S*_bP_ between pores. As it turns out, only the term *D*/Δ*S* will appear in the solution of the diffusion problem posed in Figure 18, with Δ*S* = *S*_bP_ - *S*_Pt_ (and *S*_bP_ > *S*_Pt_) for normal pore growth. A complete theoretical impedance for bsi-IS relevant for n-macro(aqu, bsi) pore etching is given in Equations (5) and (6):

(5) $$Z_{\text{complete}}(\omega ,d_{B},L,\Delta S_{P})=A_{1}\left(Z_{\text{bsi}-\text{1}}+\frac{A_{0}}{\frac{1}{Z_{\text{bsi}-\text{1}}}+A_{2}\sqrt{i\omega }}\right)\left(\frac{\frac{1}{\sqrt{\frac{1}{L^{2}}+\frac{i\omega }{D}}}}{\frac{1}{\sqrt{\frac{1}{L^{2}}+\frac{i\omega }{D}}}+\Delta \frac{D}{S_{p}}}\right)$$

(6) $$Z_{\text{bsi}-\text{1}}(\omega ,d_{B},L)=\frac{\text{d}j_{\text{sem}}(\omega ,d_{B},L)}{dP(\omega )}\propto \frac{1}{\cosh\left(l_{\text{Pore}}\sqrt{\frac{1}{L^{2}}+\frac{i\omega }{D}}\right)}$$

This does not look too simple, but it is just as complex as it has to be. Three contributions can be distinguished: the first and the second large bracket in Equation (5) and the quantity *Z*_bsi-1_ defined in Equation (6). The latter is the complete “simple” solution for the classical “Lehmann model” (cf. Section 2.3 or [28], *i.e.*, of the diffusion problem, where a given number of holes is generated at the specimen’s back side by illumination, diffuses through the sample with a diffusion coefficient *D*, and recombines either in the bulk (described by the diffusion length *L*), or is converted to current at the pore tips. The later process is mathematically described by assigning an interface recombination velocity *S*_P_ = ∞ (or rather 1/*S*_P_ = 0) to a fictitious interface defined by the pore tips as shown in Figure 18. Within the SCR model this should be a reasonable approach, because all holes reaching the position of this fictitious interface would end up at a pore tip and thus disappear.

**Figure 19:**
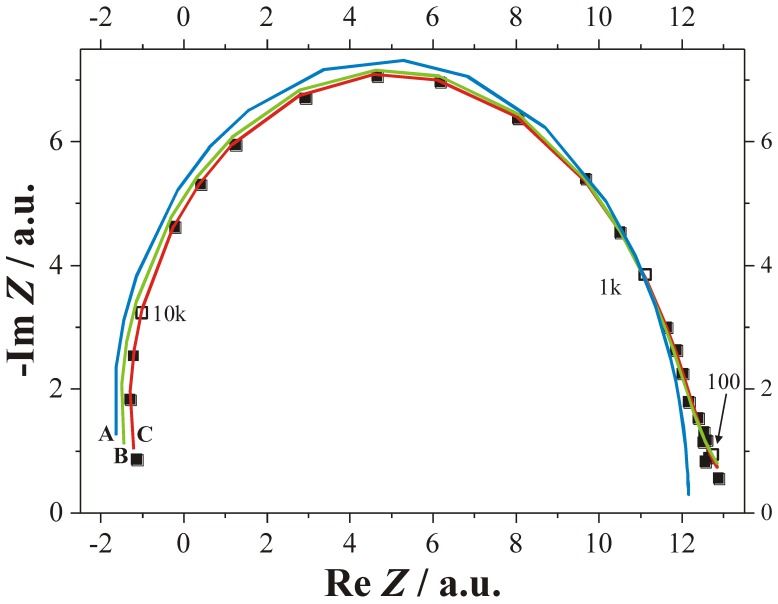
Fitting theory to measured *in situ* bsi-IS data (black squares). Curve A (blue) results from Equation (6) only. Curve B (green) results from Equation (5) without the first term, and curve C (red) is based on the complete impedance as given in Equation (5). Numbers indicate some of the 27 frequencies used in Hz (total range 10 Hz - 40 kHz).

However, as shown in Figure 19, where a measured spectrum is printed in a conventional Nyquist plot (the (negative) imaginary part of the impedance is plotted *vs.* the real part), matching bsi-IS data against just Equation (6) alone does not produce a perfect fit. In conclusion, the SCR-model for n-macro(bsi) pore growth is too simple and must be augmented. As outlined in [28] in more detail, the problem is that the tip of the macropore is covered with oxide most of the time and thus cannot process holes at any time and at any place.

At a next step of sophistication, we now assume that some holes arriving at the depth *l*_Pore_ of the pores, do not all contribute to the pore tip current, but are also consumed at areas not very close to the pore tip; *i.e.*, at pore walls. Mathematically this is expressed by changing the boundary condition with respect to the interface recombination velocities to a periodic expression, where the recombination velocities between the pore tip and the point halfway between the pores differ by some Δ*S* as outlined above. Taking this into account, the solution of this diffusion problem gives as a second approximation *Z*_bsi-1_ times the second bracket in Equation (5), that contains as a new parameter *D*/Δ*S*. The match to all the experimental data is better now, as shown in Figure 19, and in a large number of other data not shown (note that just one pore etching experiment yields several 1,000 bsi-IS spectra), but still not perfect.

A perfect match can be obtained in a third step, if one assumes that some fraction of the holes reaching the pore tip region is not immediately consumed at the tip, but has to “wait” a bit, meanwhile diffusing around. In physical terms this means that oxide covered parts of the pore tip cannot process holes instantly, but need to wait until the oxide thickness has been sufficiently reduced. More succinctly stated (and with knowledge of quantitative data, some of which are shown below), at this point it becomes necessary to postulate that the pore tip is actually covered with (thin) oxide most of the time.

The proper term for this “waiting time” presently cannot be obtained from solving an appropriately posed diffusion equation, but is derived by adding a general diffusive term akin to what is known as “Warburg impedance” in *VI*-IS [149]. The quotation marks refer to the fact that “proper” impedances are measured in Ohm [Ω], as noted before. Besides the parameters *L*, *l*_Pore_, and *D*/Δ*S*, two new parameters, *A*_0_ and *A*_2_, emerge beside *A*_1_, that is just a proportionality constant, and thus simply a measure for the over-all etching area defined by the envelope of the pore tips, including the area of under-etching at the O-ring or under masks. The combination of *A*_0_ and *A*_2_ quantifies the fraction of that part of the etching current that experiences the additional diffusion around the pore tips.

Evaluating the *in situ* FFT bsi IS data thus should give *in situ* information about the pore depth *d*_Pore_, the diffusion length *L*, the total active area *A*_1_ (essentially reflecting the degree of under etching), the detailed current flow processes around the pore tips quantified by *A*_0_, *A*_2_, and *D*/Δ*S*, and the growth speed *v*_tip_ of the pore *via* *v*_tip_ = d*d*_Pore_/d*t*. Since *L* is a constant by definition, the numerical evaluation procedure determines *L* from the first few spectra and then takes it as constant, making subsequent data extraction easier and more precise. Since *l*_Pore_(*t*) is now a known quantity, all the other parameters extracted can be rescaled from a function of time to a function of pore depth, if so desired.

In the case of the *VI*-IS, there is no choice but to find the best fit to equivalent circuits expressed either in suitable circuit diagrams containing capacitances, resistors, and Warburg impedances, or slightly more abstract, but fully equivalent, by using model equations describing processes with as many time constants as needed; cf. e.g., the standard text book of MacDonald for details [149]. The equation that matches many thousands of *in situ VI*-IS obtained so far rather well is:

(7) $$Z_{U}(\omega )=R_{s}+\frac{1}{\left(\frac{i\omega \tau_{slow}}{\left(R_{p}+\Delta R_{p})(1+i\omega \tau_{slow}\right)}+\frac{1}{R_{p}(1+i\omega \tau_{slow})}\right)+i\omega C_{p}}$$

Following the standard interpretation of such a model equation, *R*_s_ describes the ohmic losses due to a general series resistance, *C*_p_ describes the capacitance of the interface, and *R*_p_ the so-called chemical transfer resistance of the chemical dissolution process. The chemical dissolution splits up into two processes with different reaction rates, characterized by the relaxation time *τ_slow_* of the slow process, and the time constant *R*_p_*C*_p_ of the fast process. The difference Δ*R*_p_ describes the increase in the chemical transfer resistance at higher frequencies. A similar approach has been used successfully for unravelling pore formation in InP, cf. [153,154] for details.

The processes in question can only be the current-driven direct dissolution of Si (typically occurring with a valence *n*_dd_ = 2) and current-driven Si oxidation (valence *n*_Ox_ = 4) together with the purely chemical dissolution of the oxide, as detailed in Section 2. Assuming that direct Si dissolution is the “fast” process, and that Si dissolution by oxide formation plus oxide dissolution is the “slow” process, it is possible to derive an equation for the over-all valence *n* (for a more detailed derivation see [28]):

(8) $$n=\frac{4}{2-\frac{\Delta R_{p}}{R_{p}+\Delta R_{p}}}$$

A full evaluation of *in situ* FFT *VI*-IS data thus provides as a function of time (or pore depth) the following parameters: *R*_s_, *R*_p_, *C*_p_, *τ_slow_*, Δ*R*_p_, and as a combination of those parameters the valence *n* calculated from Equation (8).

Summing up, *in situ* multimode FFT IS allows keeping track of 12 parameters that encode more or less directly properties of the Si (e.g., its diffusion length *L*), the actual pore geometry (e.g., the pore depth *l*_Pore_, or the degree of under-etching at O-rings or masked parts of the sample *viaA*_1_), the processes at the interface (e.g., the valence *n* or Δ*S*), plus some “electrical” properties like the series resistance and interface capacitance, and thus crucial information not only for controlling pore growth, but for any modeling effort that endeavors to obtain a better understanding of the process.

It is beyond the scope of this section to discuss in detail what kind of conclusion with respect to the pore etching process can be drawn from multi-mode *in situ* FFT IS, and we will restrict ourselves to two examples of particular interest for the next sections.

### 4.2. Selected Results from Si Pore Etching

There are just a few investigations using the full power of dual-mode *in situ* FFT IS at present, but they have already yielded important data, cf. [28]. In particular, it could be shown that n-Si-macro(bsi, litho) pore etching under “standard” conditions, *i.e.*, aqueous HF electrolyte with an HF concentration around 5%, proceeds rather smoothly, *i.e.*, the parameters extracted from the IS data change smoothly (*i.e.*, the pore depth) or only very little (e.g., the valence *n* or the pore growth speed *v*_tip_ = d*d*_Pore_/d*t*), this is not the case for the viscous and/or HAc bearing electrolytes that allow faster and deeper macropore growth [140]. The system shows what could be termed “chaotic” behavior. The intentional use of the word “chaotic” is meant to imply that deterministic chaos might be found under those conditions, but that has not yet been proven.

**Figure 20:**
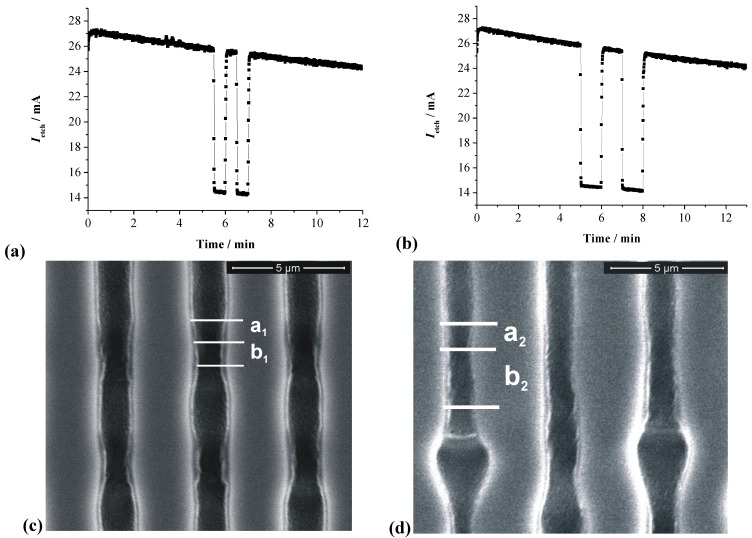
SEM cross-section of n-Si-(bsi, 5 Ωcm, litho, aq. 15 wt % HF + 0.42 g/L of CMC) macropores c) and d). After 5 min the current was modulated stepwise two times, with different periods: 1 min and 2 min as shown in a) and b), respectively. The white lines indicate the borders of regions a and b as described below and in Figure 21 and Figure 22.

Here we restrict ourselves to an example that relates to the rich pore structures shown in Figure 20. Typical “fast” n-Si-macro (bsi, 5 Ωcm, litho pre-structured, aq. 15 wt % HF + 0.42 g/l of CMC) pores were grown under conditions similar to those shown in Figure 13, except that the current was not modulated most of the time, but only for two periods as shown schematically in Figure 20(a) and (b).

**Figure 21:**
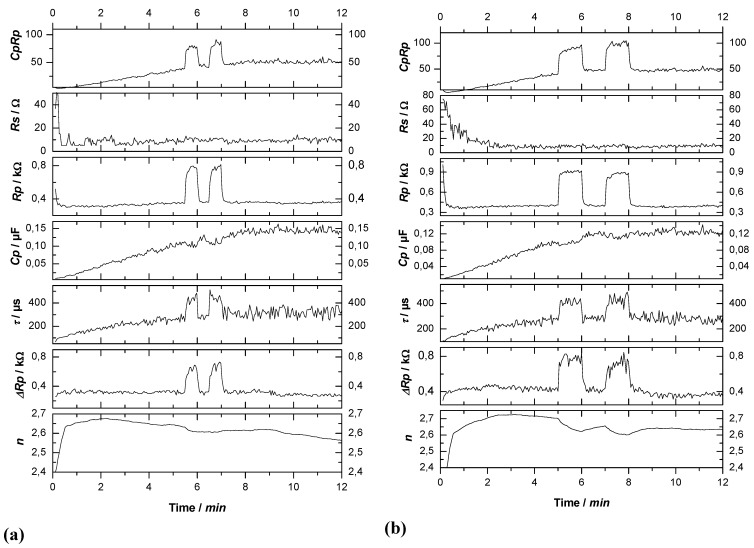
Etching parameters as obtained from the fit of the impedance data for the macropores shown in Figure 20.

**Figure 22:**
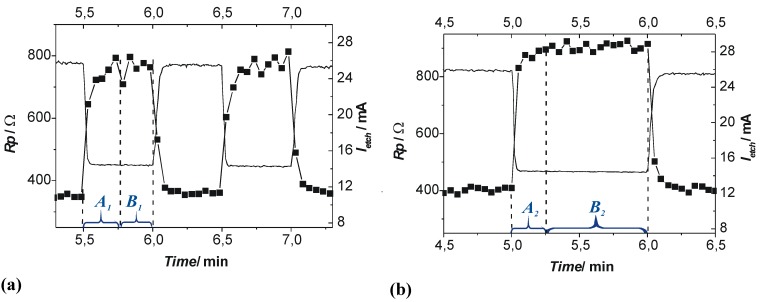
Details of the transfer resistance *R_p_*, as presented in Figure 21.

Figure 21 shows the most interesting parameters extracted from the impedance measurements for this case. While the series resistance *R_s_* and the interface capacitance *C_p_* do not change significantly upon changing the current density, strong changes are found for the time constants *C_p_R_p_* and *τ*, as well as for the transfer resistances *R_p_* and Δ*R_p_*. The time constants *C_p_R_p_* and *τ* show a significant dependence on time, resp. pore length, while the transfer resistances *R_p_* and Δ*R_p_* are nearly independent of the pore length. Since the noise in *R_p_* is much smaller than the noise in Δ*R_p_* and the changes in the transfer resistance *R_p_* are most easily understood; a detailed view is shown in Figure 22 (note the factor 2 in the scaling of the time axis).

*R_p_* should scale inversely proportional to the integral pore tip area, *i.e.*, the actively etched area. In the case of n-macro(bsi) pores the etched area is the sum of all macropore tips, which under perfect conditions is proportional to the current density. Figure 22(a) and Figure 22(b) both show an increase of *R_p_* by roughly a factor of two after reaching steady state between the phases of high current density and of low current density. This is in good agreement with the measured decrease of the pore tip areas of a factor of two.

Steady state is reached for both experiments after roughly 0.5 min; this is marked by dotted lines that separate the areas "A_1_" and "B_1_" in Figure 22(a) and "A_2_" and "B_2_" Figure 22(b). A very good agreement to the corresponding areas in Figure 20 is found: the horizontal lines mark the starting point and the end point of the areas "a_1_" and "b_1_"in Figure 20(a) and "a_2_" and "b_2_" in Figure 20(b). A nearly linear transition from large pore diameter to small pore diameter is found. The areas "a_1_" an "b_1_" in Figure 20(a) correspond to the areas "A_1_" and "B_1_" in Figure 22(a) [the same can be observed for Figure 20(b) and Figure 22(b)].

We will not go into more details here (cf. [155] for this), but only point out that multi-mode *in situ* FFT IS allows indeed to “look” at pore etching processes in considerable detail and that it will, in good time, help to understand pore etching. While at present, for the case study presented in Figure 20, Figure 21 and Figure 22, just as many questions are raised as are answered, definite progress will be made if pore formation models can explain these experimentally observed salient features.

### 4.3. A Case Study for InP and GaAs

#### 4.3.1. Studying Crysto and Curro Pores in InP

**Figure 23:**
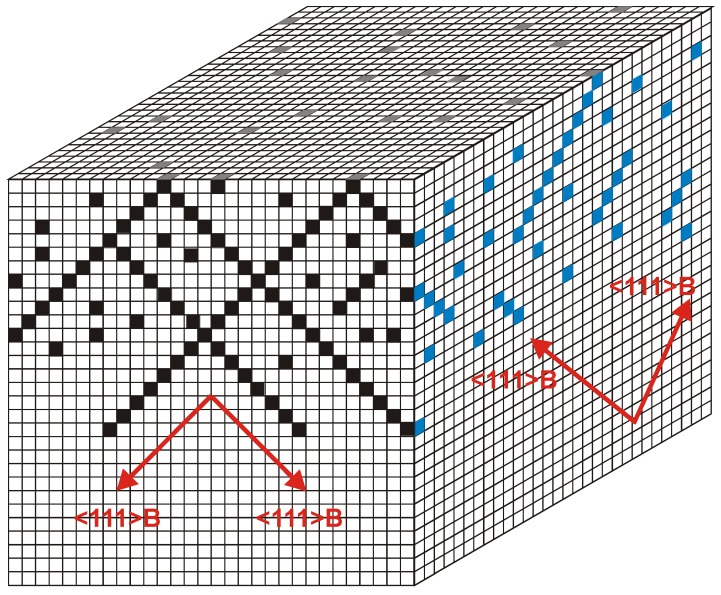
Three dimensional Monte Carlo model for crysto pore growth in InP and GaAs.

In [136,154] a quantitative model for crysto pore growth in InP and GaAs was presented that is reproduced in Figure 23. The model utilizes a Monte-Carlo approach; it is of the stochastic current-burst type. In essence, an array of (1,024)^3^ = 1.07 × 10 ^9^ voxels has been used, with a mesh size set to 100 nm, corresponding to the experimentally determined typical n-InP-macro(crysto) pore size. Some distribution of nucleation sites serves as starting condition. Pores can only grow in the <111>B directions, as indicated in Figure 23; in each time step a suitable voxel is “etched out”. The key point of the model is that it allows pore branching by assigning two different branching probabilities *k_tips_* and *k_walls_*, respectively, to branching at a pore tip or from a pore wall. In addition, an adjustable length of the pore of the order of the space charge region (SCR) width *d*_SCR_ is assigned *k* = 0, *i.e.*, it will not be able to form branches on that length, because as long as the SCRs overlap the new tip cannot experience the full voltage drop. In a last point it is assumed that all pores stop to grow, if other pores block their trajectory. In other words, pores cannot grow through the space-charge-region surrounding other pore walls.

**Figure 24:**
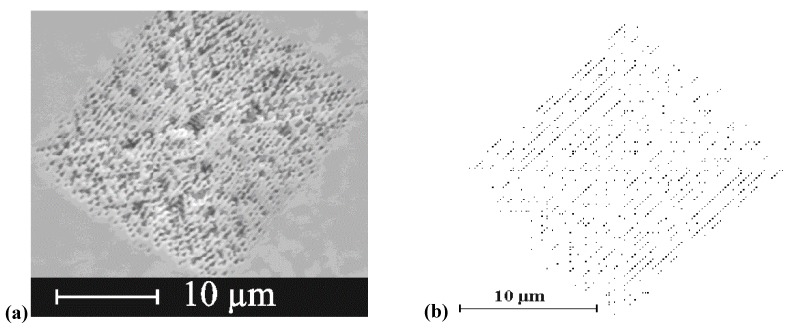
Crysto pore domains in GaAs. (a) SEM surface view of a crysto pore domain. Constant current experiment: *j* = 80 mA/cm^2^, electrolyte concentration 5 wt % HCl aq. (b) Corresponding results from the Monte-Carlo-simulations.

Running the model with a suitable set of branching probabilities reproduces many experimentally determined features of n-InP/GaAs-macro(crysto) pores, Figure 24 shows an example.

Running *in situ* FFT IS while etching n-InP-macro(crysto or curro) pores is possible and produced a wealth of data, which, together with a model briefly described above and suitable equivalent circuits for the IS data, has greatly enlarged the understanding of these pores.

{100}-oriented single-side polished n-type InP wafers with a doping concentration of *N_D_* = 1 × 10^17^ cm^-3^ were used for experiments in an electrochemical double-cell described in detail in [55]. Aqueous HCl with a concentration of 5 wt % (= 1.4 M) has been used as electrolyte. All experiments have been performed in the galvanostatic mode at a current density of *j* = 0.4 mA/cm^2^, on a sample area of *A* = 0.25 cm^2^, and at a constant temperature of 20 °C. To achieve a better nucleation, experiments were started by a current pulse of 40 mA/cm^2^ for 1 s. Several experiments have been performed for different etching times between 5 min and 360 min. The resulting pore structures have been investigated by scanning electron microscopy (SEM).

**Figure 25:**
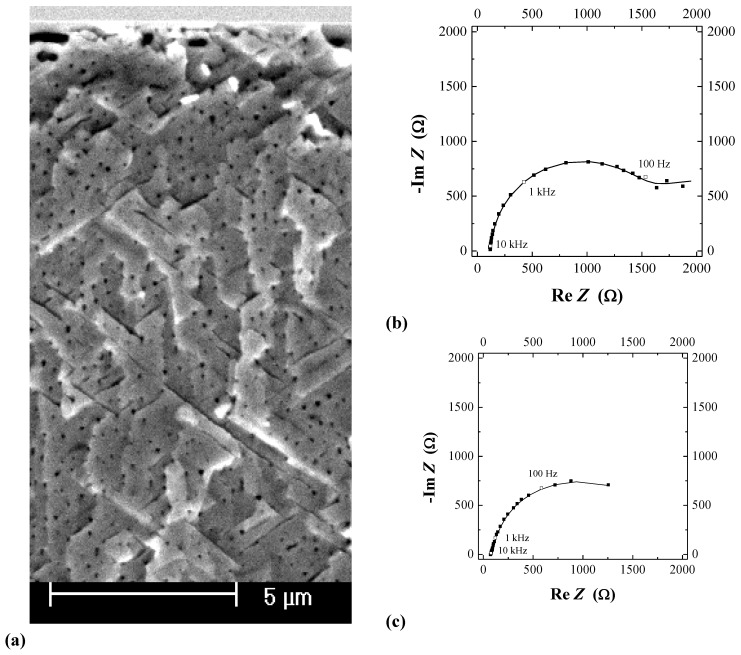
(a) Typical cross-section of n-InP-macro(crysto, HCl) pores. (b) and (c) Nyquist plot of IS spectra obtained during the experiment at *t* = 7 min (b) and 75 min (c).

Figure 25 shows a typical crysto pore structure obtained together with two Nyquist plots (out of ≈ 15.000) obtained after 7 min and 75 min, respectively.

**Figure 26:**
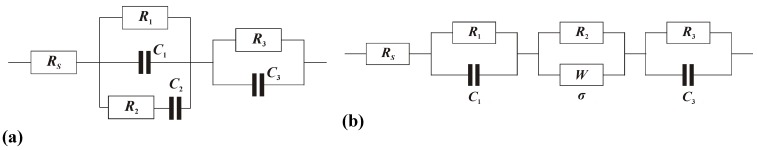
(a) Equivalent Circuit model of the impedance during crysto pore growth in n-type InP. (b) Equivalent Circuit model of the impedance during curro pore growth in n-type InP.

All IS data match extremely well with simulated data from the equivalent circuit shown in Figure 26 a). Matching IS data to an equivalent circuit is the best one can do at present since the theoretical *I*(*U*) characteristics and therefore also the impedance *Z*(*ω*) = d*U*/d*I* is what one is after, as in the Si case outlined above. Note that with less than the four resistors and three capacitors no good match is possible for all spectra obtained.

The resistors and capacitors in the equivalent circuit diagram can be interpreted to a large extent in terms of the reactions taking place, cf. [136,154] and below.

The impedance of the equivalent circuit shown in Figure 26a) is:

(9) $$Z(\omega )=R_{S}+\frac{1}{\frac{1}{R_{1}}+i\omega C_{1}+{\left(R_{2}+\frac{1}{i\omega C_{2}}\right)}^{-1}}+\frac{R_{3}}{1+i\omega R_{3}C_{3}}$$

Of particular interest are the related three time constants:

*τ*_1_ = *R*_1_*C*_1_, *τ*_2_ = *R*_2_*C*_2_, and *τ*_3_ = *R*_3_*C*_3_ (10)

**Figure 27:**
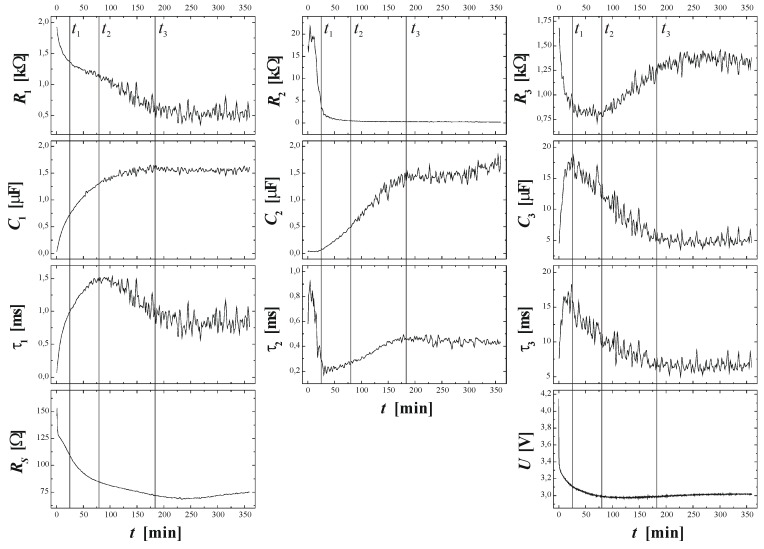
Time dependence of the fitting parameters contained in Equation (9) and the voltage *U*(*t*). The time constants *τ*_i_ result as the product of the respective resistance *R*_i_ and capacitance *C*_i_.

This model function contains seven parameters, and we can identify *R*_S_ with the serial resistance, *R*_1_, *R*_2_, and *R*_3_ with chemical transfer resistances; with the corresponding capacitances *C*_1_, *C_2_*, and *C*_3_. The values of the by now (7 + 3) variables (including the time constants) can be found by matching measured data against Eq (9) on-line. One plot of all data *vs.* time (or about pore depth) is shown in Figure 27.

The time dependence of the 11 parameters as shown in Figure 27 exhibits three characteristic points *t*_i_ in time, which are marked with dotted lines in Figure 27. At *t*_1_ the slopes of the three resistances get drastically smaller, and extreme values for the time constants *τ*_2_ and *τ*_3_ are found. The time *t*_1_ at roughly 20 min coincides well with the time (= depth) where pores growing upwards are first observed.

The most significant time *t*_3_ in this context is reached after about 180 minutes. From *t*_3_ on most of the curves do not change significantly anymore and thus stay constant. This indicates that pore growth has reached a steady state, *i.e.*, the pore structure is qualitatively the same between the pore tips and the top layer of highest density. The slight linear increase of *R*_S_ is in good agreement with these results, since in a steady-state of pore growth only *d*_pore_ changes, and thus the linear change in *R*_S_ can be attributed to the linearly increasing ohmic losses. Without going into details (cf. [136,154] for this), this is in good agreement with the *ex-situ* results [cf. Figure 25(a)] for one example for etching times larger than 200 min.

A third transition time *t*_2_ occurring after roughly 80 min is clearly visible in the curves *R*_3_, *R*_1_, and *τ*_1_. It is obvious that after a certain pore density has been reached, the upward growing pores must grow into regions where many pores are already growing downwards. These pores thus will have to stop growing, as e.g., shown for GaAs as well [14,58]. The time *t*_2_ could well be related to this process, *i.e.*, it indicates a time when a significant number of upward growing pores stop, thus paving the path to steady state.

A similar approach with n-InP-macro(curro) showed that a different equivalent circuit, as presented in Figure 26(b), was needed to match the measured impedance results. The impedance of the equivalent circuit shown in Figure 26(b) and the time constants going with it are:

(11) $$Z(\omega )=R_{S}+\frac{R_{1}}{1+i\omega \tau_{1}}-\frac{R_{2}}{1+(1+i)\sqrt{\omega \tau_{2}}}+\frac{R_{3}}{1+i\omega \tau_{3}}$$

*τ*_1_ = *R*_1_*C*_1_, *τ*_2_ = *R*_2_^2^*/2σ*^2^, and *τ*_3_ = *R*_3_*C*_3_ (12)

This model corresponds to an equivalent circuit consisting of a serial resistance *R_S_* in series with two time constants *τ*_1_ and *τ*_3_, in series with a Warburg impedance in parallel to a resistor, characterized by the parameters *τ*_2_ and *σ*, in parallel to the resistance *R*_2_. *R*_1_, *R*_2_, and *R*_3_ describe the respective transfer resistances. Different models with 7 or less fit parameters have been tried as well, but yielded only unsatisfactory results, thus lending credibility to the applied fitting model.

**Figure 28:**
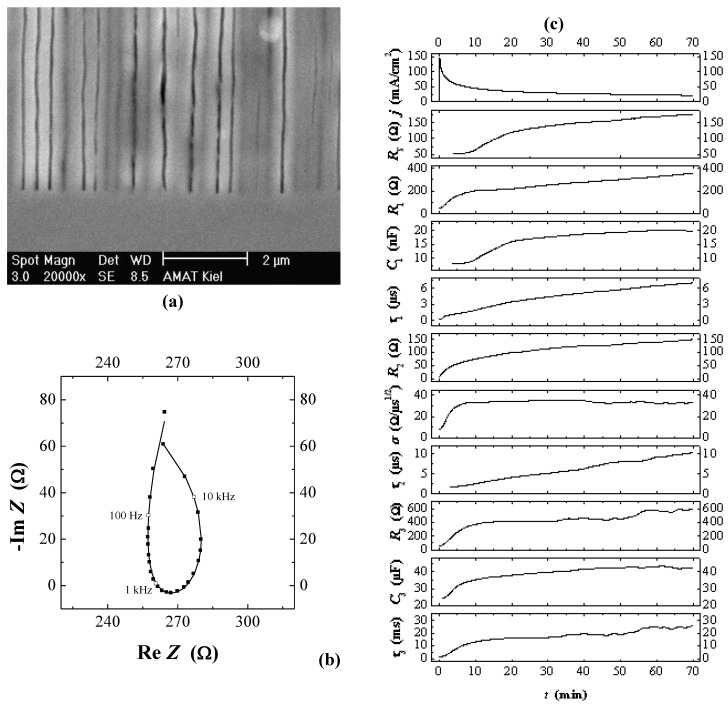
(a) Curro pores in (100) n-type InP, *N*_D_ = 1 × 10^17^ cm^-3^. (b) A typical Nyquist plot of the impedance spectrum recorded during curro pore etching (dots: measured data, line: fit according to Equation (11)); frequency range 100 Hz–30 kHz (increasing counter-clockwise), and **(c)** the resulting time dependence of the fit parameters.

Since the impedance measurements have been performed *in situ* during the pore growth every 1.5 seconds, the aforementioned fitting parameters can be plotted as a function of time. The resulting curves are shown in Figure 28(c) for a 70 min curro pore etching experiment, together with an SEM cross section of the pores produced [Figure 28(a)], and one Nyquist plot of a measured impedance spectrum together with the fitting curve [Figure 28(b)].

As before for the Si case and the InP crysto pores, a number of new and far-reaching conclusions can be drawn from the curves in Figure 28. We will, however, not subject the reader to a necessarily quite lengthy discussion of what exactly the measured data imply but refer to [153] and forthcoming publications in this context. Instead, we present recent new data and extension of our model.

**Figure 29:**
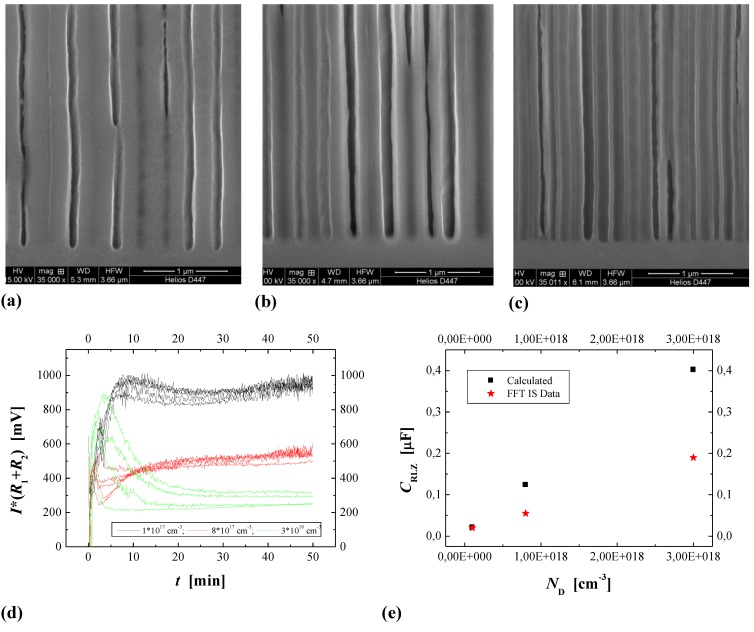
Curro pores etched in (100) n-type InP with different doping concentrations: (a) *N*_D_ = 1 × 10^17^ cm^-3^, (b) *N*_D_ = 8 × 10^17^ cm^-3^, and (c) *N*_D_ = 3 × 10^18^ cm^-3^. (d) Product of the current *I* and the sum of the two transfer resistances *R*_1_ and *R*_2_. Different curves for each doping concentration represent differing etching potentials. For details see text. (e) Space charge capacitance, as calculated from theory (black boxes) and measured by FFT IS (red stars).

Figure 29(a-c) show n-InP-macro ({100}, curro, potentiost.) pores for three different doping concentrations *N*_D_ etched under potentiostatic conditions. For each doping concentration exists a rather broad window of etching potentials in which “good quality” curro pores can be grown, *i.e.*, rather straight pores with smooth walls growing self-ordered in a hexagonal lattice, as described in [119] and shown in Figure 7(a). The aforementioned potential ranges have been estimated to be 6 V to 8 V for *N*_D_ = 1 × 10^17^ cm^-3^, 4 V to 7 V for *N*_D_ = 3 × 10^17^ cm^-3^, and 2 V to 4 V for *N*_D_ = 3 × 10^18^ cm^-3^. In these potential windows, the pore morphology does not change significantly, *i.e.*, pore diameter and pore wall thickness stay constant. A comparison of the pore morphology for the different doping concentrations yields an additional and surprising result: the pore diameter is not dependent on the doping concentration, as one would expect from results for n-Si-macro/meso(breakdown) pores given in [2,19]. Only the pore wall thickness changes as expected, since it is generally accepted that this value corresponds roughly to twice the thickness of the space charge region *d*_SCR_, which is proportional to *N*_D_^-0.5^.

Figure 29(d) shows the product of the etching current *I* and the sum of the two transfer resistances *R*_1_ and *R*_2_, which have been measured by FFT IS. Different colors indicate the different doping concentration, and individual curves for one color represent the different etching potentials used inside the respective “potential window” given above. The product *I*(*R*_1_ + *R*_2_) has the dimension of a voltage [V] with rather similar values for all external potentials within the respective potential windows. Its value also hardly changes with time after the nucleation phase has been finished, and it depends (only) on the doping concentration.

In our model this voltage value has been interpreted as the internal potential *U*_int_ = *I*(*R*_1_ + *R*_2_) that drops in the space charge region (SCR) of the semiconductor electrode, where *R*_1_ and *C*_1_ represent the SCR transfer resistance and capacitance, respectively. *R*_2_ is then an additional resistance that describes the frequency dependence of the avalanche breakdown process, which is the commonly accepted hole generating process during pore etching. It is noteworthy that *U*_int_ defined in this way is different from the externally applied potential minus the ohmic losses as expressed in Equation (4) (it is considerably smaller) and that it can be used to obtain more information. In particular, based on this interpretation of the FFT IS data, it was possible to compare data measured by FFT IS with theoretically calculated values for three quantities:

1) With the potential *U*_int_ the thickness of the space charge region *d*_SCR_ can be calculated for the specific pore front geometry encountered (cf. [156]). The resulting values match the measured values (half of the pore wall thickness) very well.
2) With the potential *U*_int_ the capacitance of the space charge region *C*_SCR_ can be calculated for the specific pore front geometry encountered assuming semi-spherical pore tips. The result matches well with the values for *C*_1_ as directly measured by FFT IS; this is illustrated in Figure 29(e). The somewhat higher values of the calculation for higher doping concentrations might be caused by significant deviations of the pore tip shape from an ideal semi-sphere. This changes the active area and thus *C*_1_.
3) With the potential *U*_int_ the breakdown field strength of the avalanche process can be calculated, again with the same geometrical considerations as for *C*_1_. A comparison with theoretical values [157] for solid pn-junctions shows that the values obtained in this work are somewhat smaller as one would expect and show the correct dependence on the doping concentration *N*_D_.

The model also allows for a simple physical interpretation of the negative resistance *R*_2_ [the “inductive” loop in Figure 28(b)]. It represents the impedance of the avalanche breakdown mechanism, which is known from theory (cf. e.g., [128]) to show negative impedance since its *IV* characteristics contain parts with a negative slope and thus a negative differential resistance.

Our model thus allows for the *in situ* extraction of several essential etching parameters, like the internal potential present at the interface, the breakdown field strength of the avalanche process, or the capacitance of the SCR. These parameters are either not accessible by *ex-situ* measurements, or the measured parameters do not reflect the situation which is present during pore etching (= current flow = non-equilibrium) and are therefore not relevant. Since the model is rather recent, future work should yield further insight into the details of the pore etching mechanism for curro (and crysto) pores. In particular, experiments at the edge of the “potential window” suitable for pore etching should yield results on why pore growth becomes unstable or even impossible.

#### 4.3.2. Crysto and Curro Pores in GaAs

So far only crysto pores have been observed in GaAs [14]. This can be understood to some degree if one assumes that the surface passivation of GaAs is very pronounced, cf. [14]. In the context of the generalized pore formation model championed here, some predictions with respect to GaAs pores can be made: first, it should be possible to induce curro pores in GaAs, if the conditions are right. Second, the model presented above for InP including the FFT IS data, should also be applicable to the GaAs case, if the differing passivation behavior is taken into account. Very recent results reported here for the first time seem to indicate that both predictions are true. Experiments were performed in standard equipment as described before or in [14], the specimen used were {100} n-GaAs, Te doped, doping levels ranging from (2-3) × 10^15^ cm^-3^ to (4–11) × 10^17^ cm^-3^.

**Figure 30:**
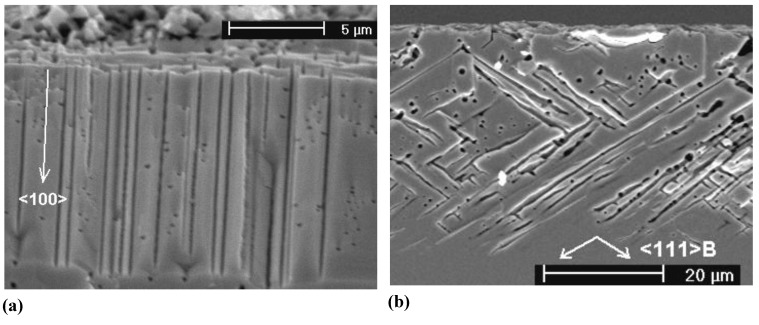
Pores in GaAs. a) First curro pores. b) Relatively uniform distribution of crysto pores.

The “right conditions” alluded to above include foremost rather homogeneous pore growth - and this is difficult to achieve in GaAs. If pore growth is not homogeneous (for example, if just a few big pores are formed instead of many uniformly distributed ones), neither a typical pore structure can be deduced from *ex-situ* SEM pictures, nor is it possible to evaluate FFT IS data (cf. [28]). Uniform pore distributions are obtained if one or both of the following criteria is met: i) The initial nucleation is uniform with a density matched to the typical pore geometry; ii) Non-uniformly nucleated pores branch rapidly and repeatedly, leading to a uniform distribution some µm below the original surface (cf. Figure 30b) for example). GaAs normally does not meet both criteria. Initial nucleation is a complex and tortuous process [49], and branching of pores is rather rare, because of the strong passivation. Nevertheless, rather homogeneous nucleation could be achieved now by a mix of measures reminiscent of what has been reported in the case of Ge [52], and progress beyond the known state-of-the-art was possible.

First, curro pores as shown in Figure 30(a) have been observed for the first time. Uniform crysto pore growth is a prerequisite for that; Figure 30(b) shows an example of the uniformity achieved at present. Note that some older GaAs pictures appear to show more uniform pores (e.g., in Figure 3), but these pictures showed only a small part of the specimen while Figure 30(b) is representative of the whole sample.

**Figure 31:**
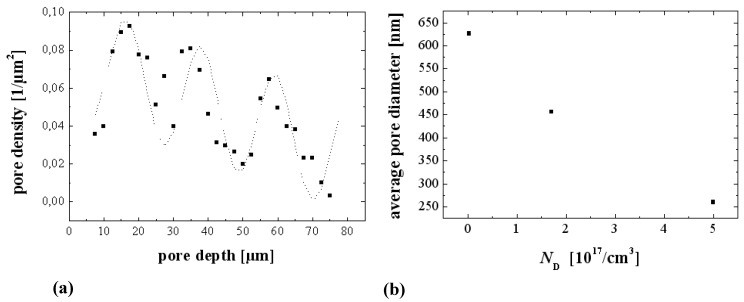
Some quantitative data with respect to GaAs crysto pores. a) Pore density as a function of pore depth for a doping level of *N*_D_ = (2-3) × 10^15^ cm^-3^. The line is not a fit but only to guide the eye. b) Average pore diameter as function of the doping density *N*_D_ of the n-type GaAs samples.

First valid FFT IS spectra were obtained for crysto pores. First evaluations were consistent with the approach taken for InP, provided that the branching probabilities are quite smaller than those used for modeling pore growth in InP (see above), but we will not belabor this point here. Of direct interest are the quantitative data shown in Figure 31. A plot of the crysto pore density *vs.* pore depth in Figure 31(a) shows strong oscillations, as expected from the InP model [136,154]. Since these oscillations mirror nucleation conditions, it is not surprising that they are more pronounced in GaAs than in InP. For the first time some data for the correlation between crysto pore diameters and doping has been measured; the result is shown in Figure 31(b). The diameters decrease with increasing doping as expected for crysto pores. Note that for curro pores this is different as outlined above.

Far more details could be reported, but the point is made once more. Some “meta” principles are indeed involved in pore formation that transcends the detailed and specific chemistry of dissolution.

## 5. Some Applications of Porous Semiconductors

### 5.1. General Remarks

The electrochemistry of Si, while fascinating enough from a basic science point of view, rarely was studied without some kind of application in mind. Even before the advent of luminescent microporous layers [27,67,68,129,158] and deep n,p-Si-macropores [66,159] some applications were proposed or even investigated in detail. For example, the "FIPOS" process (short for "full isolation by oxidized porous silicon" (cf. [160]), exploited the selective formation of microporous Si in p-type material and its quick oxidation for microelectronic device purposes. Selective anodic etching of crystal lattice defects in early multi-crystalline Si sheets intended for use in solar cells around 1980 [161,162] could differentiate between docile and harmful defects. Based on this, one of the authors (hf) together with the late V. Lehmann [163] used Si electrochemistry to characterize early solar Si samples, which lead not only to the discovery of n-Si-macro(bsi, aqu, litho) pores [66] but also to the “ELYMAT” (short for ELectrolYtical MetAl Tracer) technique [164,165,166]. The ELYMAT exploits the specific properties of Si-electrolyte junctions for mapping the minority carrier lifetime in Si wafers; its development culminated in a successfully marketed piece of equipment capable of producing lifetime maps of Si wafers. 

Several thousand publications concerning porous Si have been published since 1990, and many of these papers mention some applications or describe it in considerable detail. Just sorting out the major product lines envisioned would go far beyond the scope of this review, and it is impossible to go into the sometimes quite involved details of some specific applications. Instead, only a brief and somewhat subjective overview of some general developments will be given, followed by a somewhat more detailed description of very recent (and not yet fully published) new developments in the environment of the authors.

There are several reviews and books emphasizing application of porous Si [2,13,112,167,168], in [11,169,170,171,172,33] mostly optical applications of porous Si are covered. Whereas in [173,174] the topic “photonic crystals” is prominent or, somewhat simplified, light propagation perpendicular to pores in a specific array, in the most recent additions to this topic many modes of light propagation mostly in the pore direction are covered in detail in [11,33,167].

The reader who is not too familiar with porous semiconductors should be aware of some not immediately obvious issues in the context of applications:

i) There are many potential applications where it does not matter that the porous material is actually a semiconductor. In other words, there are many applications outside the typical semiconductor domains of electronics and optics or optoelectronics.
ii) Practically all applications discussed so far use porous Si. Microporous Ge might find applications [30,175] but so far very little has been proposed with respect to applications of porous III-V’s [176,177].
iii) Some proposed applications, while perfectly viable, are rather impractical. For example, mono crystalline or multi crystalline Si solar cells come off the production line at a rate of about 1 (large) solar cell per second, leaving 1 second time for a single wafer process like pore etching. Producing many wafers in parallel would of course solve this problem but likely at prohibitive costs. Envisioning standard pore etching for some solar cell processes as proposed, e.g., in [178,179], is simply not practical. This is not to say that electrochemical processing is never useful in solar cell production; there are, in fact, considerable efforts under way to use a so-called layer transfer process involving (quick) pore etching for the production of cheap but highly efficient solar cells [180], but simply to say that economic or cost considerations cannot be neglected if one has a commercial product in mind.

While we refer the reader once more to the literature already given for a somewhat more complete overview of applications, we will finish this sub-section with a short list of particular non-obvious old and new applications of porous semiconductors, together with some promising new properties and ideas for using them. This list is subjective and somewhat biased with respect to the interests of the authors and neither comprehensive nor complete.

**Figure 32:**
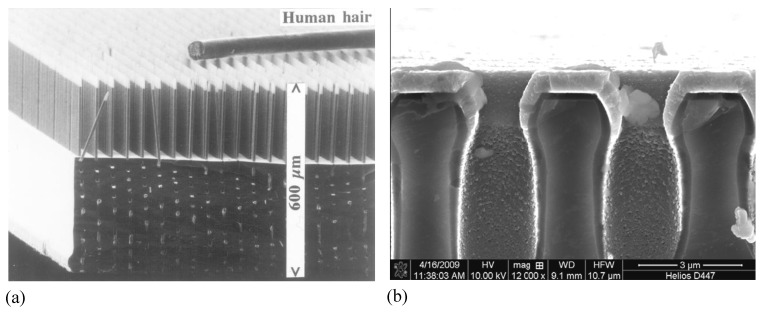
(a) Part of a 150 mm diameter X-ray filter with partially etched back Si, exposing the Pb needles filling the n-Si-macro(bsi, aqu, litho) pores [185]. (b) n-Si-macro (bsi, aqu viscous, litho) pores coated with TiO_2_ intended to be used as an UV switchable membrane for biological filtering.
4) Porous semiconductors (like other porous materials) lose whatever thermal conductivity they have, as soon as the distance between pores approaches the mean free path length of phonons. Microcrystalline porous Si is an almost perfect thermal insulator as has been demonstrated in [189,190,191]. This effect has been used for unusual kinds of loudspeakers [192] and may find future use for thermoelectric devices converting heat directly to electricity. A figure of merit for such a device is the ratio of thermal conductivity to electrical conductivity and numbers surpassing the present state of the art by a large margin should be possible with porous semiconductors. Efforts to exploit this feature are under way [193,194,195].
5) A membrane made of macroporous Si is a direct mechanical high-precision filter for particle sizes in the µm regime [196,197]. If one can induce the diameter to decrease locally to even smaller values, a sub-µm filter results, cf. [172,198] Figure 33(b). Coating the filter with e.g., TiO_2_ [cf. Figure 32(b)] adds “bio” possibilities—the filter, for example, “kills” bacteria if exposed to (ultraviolet) light [199]. Filters of this type might even be useful for brewing beer [200].
6) Biological applications of (micro and meso) porous Si have been pursued for several years by now. Not only is porous Si a very good biocompatible substrate (cell cultures thrive on it, in contrast to bulk Si [201,202,203]), porous Si implants that have been loaded with some drug before they are administered, might provide reliable long-term drug delivery without severe side effects [204,205,206].
7) Last, it is illuminating to mention the two major porous Si products that actually were on the market some years ago but discontinued at present. First, there was V. Lehmann’s DNA chip, based on n-Si-macro(bsi, aqu, litho) pores [207]. A pilot production was running successfully for several years but could not sustain its just tolerated “niche” status in a major micro-electronics production line. Second, the so-called ELTRAN™ (Epitaxial layer TRANsfer) process from CANON™ [208], also abandoned by now, employed Si mesopores as a “zipper” layer not unlike the layer transfer process for solar cells mentioned above [180].

1) High explosives from mesoporous Si. Two laboratories “discovered” the explosive properties of micro/mesoporous Si containing an oxidizing specimen more or less by accident (and fortunately without injuries to persons) cf. [181,182]. The mechanism is clear. The reaction Si + O_2_ ➔ SiO_2_ generates substantially more energy per kg than the explosion of TNT, and in a structure where a large percentage of all Si atoms are surface atoms the reaction can proceed rather fast. A sizeable project was undertaken to use “explosive Si” as a fuse for setting off airbags in cars [183], since it proceeds so fast that considerably more (valuable) time is available to process the sensory information, that might lead to an air bag deployment. The project did succeed in making a very reliable and easy-to-produce high explosive (we do not give the recipe for obvious reasons) but did not yet mature to production. There are also other efforts to use this effect [184]. This example provides perhaps the least intuitive application of porous Si and serves to illustrate that applications of porous Si may go far beyond the confines of traditional semiconductor uses.
2) Filters that pass X-rays in the direction perpendicular to the filter axis but not at some inclination (collimators) have been produced by filling n-Si-macro(bsi, lith, aqu) pores with Pb [185]. Filters like that are very useful for medical X-ray applications, in particular if soft tissue is to be investigated, e.g., in mammography. Working filters have been demonstrated, but no production has yet been started. We mention this old example, because it provides the by far easiest and possibly oldest technique for filling Si pores with a metal: just “dip” the porous substrate in the molten metal! Figure 32 gives an illustration. Note that other uses of porous Si for X-ray imaging are also possible by filling pores with a scintillator material [186,187].
3) In non-cubic single crystals the index of refraction is not a single number but a tensor, producing effects like birefringence [188]. Presently, advanced optics (including non-linear optics) exploiting this anisotropy must rely on what nature provides in the form of natural or laboratory-grown crystals. The effects are thus limited to “naturally occurring” numerical values of the tensor components and tensor symmetries that are always tied to the crystal symmetry. Pores in crystals break the symmetry. For example, cubic semiconductors like GaAs that contain several sets of pores [e.g., crysto pores in a {100} crystal that run along two <111> directions; cf. Figure 3, Figure 30(b), and Figure 25(a)], have a non-cubic symmetry from the viewpoint of light with a wavelength far smaller than the pore geometry. If crystals with an asymmetric pore set are produced, e.g., by cutting the specimen with an orientation somewhat off {100}, more symmetry breaking occurs and optical tensors not possible in any bulk crystals result [11]. The effect is beyond doubt, but needs yet to be demonstrated. Moreover, porous III-V crystals like GaP (and possibly also II-VI crystals) may also have very unusual non-linear properties as demonstrated in [176,177] for porous GaP, where a more than 100 fold increase of the second harmonic production was observed. This example serves to point out that so-called meta-materials can be made with porous semiconductors having properties that are so unusual that it is not surprising that they have not yet been exploited.

Considering this list, and keeping in mind that it is far from being complete and that we look back on almost 20 years of intensive porous Si research, the obvious question must now be asked: why are hardly any products out there on the market? 

The same questions could have been asked after many years of research into, e.g.,, liquid crystals, organic conductors, or electric cars. While some new discoveries result in products very quickly, it usually takes far more time than envisioned in a first wave of enthusiasm to develop a new product that is not only “working” but can be sold with a profit. A long list of problems encountered in moving porous semiconductor products from a “proof of principle” in the laboratory to pilot production could be easily compiled but we will only look at two important points in this context:

1) It seems to be no problem to process a 300 mm Si wafer with breathtaking complexity and precision (the cm^2^ of a 1 Gb memory chip, for example, sells for < $ 1) but very few people (if any) have anodically etched (deep) pores into Si wafers with the precision and reproducibility required for production. If that feat would be possible at all, the exceedingly large amount of money and time required to do so is simply prohibitive in laboratory work.
2) The pore etching process (and its *in situ* control) is still not understood to the extent needed in a production environment. To give an example: what do you have to specify in terms of the carrier life time if you were to etch n-Si-macro(bsi, aqu, litho) pores? It should be large, of course, but how large and with what kind of global tolerances? What is the lateral uniformity needed? If your specification is very tight, are you willing or able to pay the (possible large) additional price for those wafers? What do you have to specify in terms of the carrier life time if you were to etch p-Si-macro(org) pores? There are no data at present about the possible importance in this case. If it would be important, the question is: why?

It appears that at present porous Si is only used commercially as a sacrificial layer (cf. [209] during the production of a MEMS pressure sensor by Bosch), but nothing seems to have been published to that. We are confident, however, that porous Si will, in time, find its way into products and that the insights gained in recent years will be helpful for this.

### 5.2. Some New Applications

#### 5.2.1. Sensors Exploiting the Piezoelectric Properties of Porous III-V’s

A possible application for porous III-V compounds may come from the fact that they are in fact piezoelectric materials [210,211,212]. However, the deformation induced capacitive charge on the surface is more or less short-circuited by the finite conductivity of the material, and the piezoelectric properties are therefore of little use so far. Porous InP or GaAs, however, will be a rather good insulator and its piezoelectric properties—still those of a single crystal—might be used for applications. One such application may be found in a so-called multi-ferroic sensor for very small magnetic fields [213]. All that needs to be done is to fill the pores with a ferromagnetic material showing magnetostriction, e.g., Ni or “Terfenal” (an Fe—Tb compound). A magnetic field will change the dimension of the ferromagnetic material and the resulting deformation of the piezoelectric material produces a voltage that can be measured. It has been shown that sensors of this kind can detect extremely small magnetic fields and thus could find many possible applications.

**Figure 33:**
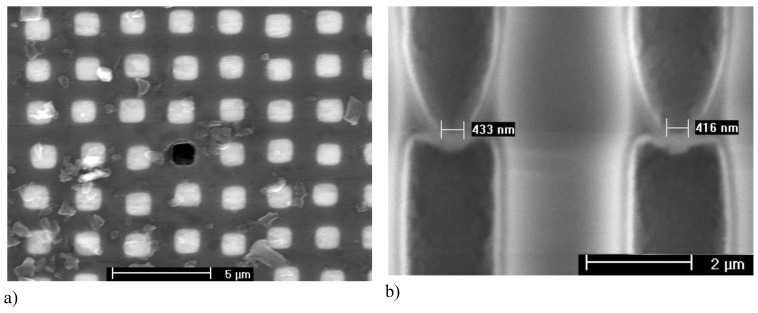
(a) Single hole with large aspect ratio in Si (cf. [220]). (b) n-Si-Macro(bsi, aqu, litho) pores with intentionally produced local constriction intended for filter usage.

#### 5.2.2. Single Holes with Large Aspect Ratios

In several branches of research, single holes with small diameters in the nm - µm range and large aspect ratios are needed but so far very hard to produce. For example, detailed studies of transport and electrochemical phenomena need holes with nanoscopic dimensions [214,215,216,217], while applications such as single-pore filtration of cells call for pores or holes with well-defined diameters of a few µm. In ref. [218] “patch clamping” is described, meaning the use of single holes with diameters around 1 µm and an aspect ratio of 200 in order to avoid laborious and difficult micromanipulations of a (micro) glass pipette under a microscope. Microfluidic applications [219] would also benefit from single holes or arrays of holes in a defined pattern and that might also be true for some nano-optics and X-ray applications. In [220] a simple way to single hole production with porous Si is described, Figure 33a) shows an example. The production process is simple: produce Si macropores, cover one pore (or some selected pores) somehow (there are many ways to do this), fill the remaining hole galvanically with, e.g., Cu; remove parts of the front and back side until the single hole formed by the unfilled pore is exposed. Single pores with diameters in the 1 µm region and aspect ratios > 100 are made with ease; diameters in the 100 nm region with comparable aspect ratios should be possible.

#### 5.2.3. Anodes for Li Ion Batteries

Applications of the Li ion battery for large-scale energy storage, e.g., for electrical cars, requires substantial improvements of the performance/cost relationship. Improvements are needed for many parameters, here we only consider capacity as measured in Wh/kg and cycle stability, *i.e.*, the number of charge / recharge cycles possible. The capacity is directly related to the amount of Li that can be intercalated in the anode (and cathode). 

It has been known for some time that Si offers one of the best weight ratios of 4,200 mAh/g, about a factor of 11 larger than for state-of-the art graphite anodes [221]. However, upon driving Li into Si, various phases like Li_12_Si_7_, Li_7_Si_3_, Li_13_Si_4_, and Li_22_Si_5_ form with a concomitant volume change of about a factor of 4 for full loading. This invariably leads to mechanical stresses large enough to fracture bulk Si into powder after the first few cycles of charging / discharging.

In [222] it was shown that Si nanowires, while doubling their diameter during the intercalation of Li, do not fracture, and that some random arrangement of nanowires with some diameter distribution centered around 89 nm can withstand more than 10 charging/discharging cycles without significant loss of capacity. Here it will be shown that using pore etching as a decisive first step can make Si nanowire anodes with superior properties.

**Figure 34:**
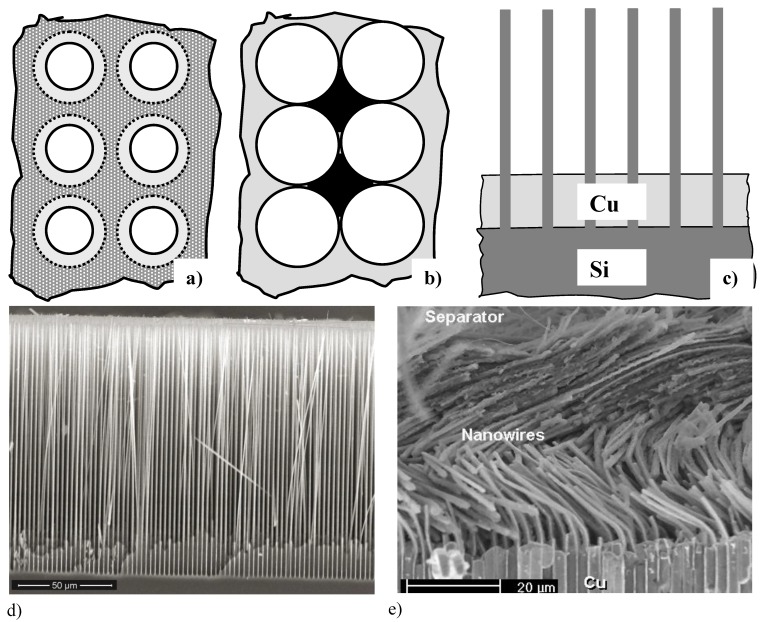
(a-c) Schematic process sequence for Cu stabilized Si nanowire anode. (d) Resulting nanowires in cross-section. (e) Anode after loading with Li and several cycles. Note that the occasional fracture of the nanowires was caused by specimen preparation.

Optimized Si nanowire anodes were made in three basic steps: 1) Electrochemical etching of p-Si-macro(litho, aqu/ethanol) pores in an optimized (lithographically determined) arrangement. 2) Uniform chemical etching of the macroporous substrate in order to increase the pore diameters to a point where the pores touch each other, resulting in nanowires. 3) Galvanic deposition of Cu onto the substrate; encasing the nanowires into Cu. This is the critical process, since it decouples the Si substrate below the nanowires from the battery and provides for efficient current extraction. Without the Cu layer, Li would also be incorporated into the bulk Si, leading to fracture and disintegration of the anode. Figure 34(a,b) illustrate these points; some details can be found in [223].

Tests are still running but more than 60 cycles have been realized without loss of capacity. The process sequence is rather simple and there are good reasons to assume that the cost of a 100 cm^2^ processed anode is in the same region as that of a solar cell, *i.e.*, approaching a few €, sufficiently low to make the anode commercially viable, too.

## 6. Summary and Conclusions

Porous semiconductors will provide a difficult yet fascinating object for research and development for many years to come. It is rather safe to say that not all manifestations of pores in semiconductors have yet been discovered and that included the well investigated case of Si and not just the more exotic semiconductors like the II-VI materials. It has been shown that many if not all pore structures are found in the intersection of electrochemistry, chemistry, semiconductor physics and the physics of critical phenomena (described by the catchwords “synergy” and “chaos”). This makes the in-depth study of porous semiconductors an interdisciplinary enterprise digging deeply into very basic research issues. On the other extreme, porous semiconductors have many potential uses that can be pursued on an empirical base without a full understanding of the intricacies of pore generation. 

Evidence was provided that pore formation includes an intrinsic stochastic component. The current burst model of the authors provides that component and is sufficient to explain some of the features observed. However, more than that is needed to understand pore formation as outlined in the context of pore growth mode transitions.

A strong case was made that deeper insights into the anodic formation process of pores requires *in situ* characterization techniques and *in situ* multi-mode FFT impedance spectroscopy (IS) was introduced as the method of choice. IS is always an indirect method in need of a model-based interpretation and it has been shown that fully quantitative *in situ* data could be obtained for cases where a good model already exists. *In situ* FFT IS, however, is still a useful tool in cases where models must yet be established and it can be predicted with confidence that it will be instrumental for many insights in the immediate future.

Porous semiconductors have not yet produced much revenue in the form of novel products despite large efforts into product development. The authors are confident, however, that novel devices will emerge and make money in due time. Several examples were given and it can be conjectured that work is also being done right now for applications not covered in this review.
